# Rapid Recall Ability of Memory T cells is Encoded in their Epigenome

**DOI:** 10.1038/srep39785

**Published:** 2017-01-05

**Authors:** Artem Barski, Suresh Cuddapah, Andrey V. Kartashov, Chong Liu, Hiromi Imamichi, Wenjing Yang, Weiqun Peng, H. Clifford Lane, Keji Zhao

**Affiliations:** 1 Divisions of Allergy & Immunology and Human Genetics, Cincinnati Children’s Hospital Medical Center and Department of Pediatrics, University of Cincinnati College of Medicine, Cincinnati, OH, 45229, USA; 2 Department of Environmental Medicine, New York University School of Medicine, NY, 10987, USA; 3 Clinical and Molecular Retrovirology Section, Laboratory of Immunoregulation, National Institute of Allergy and Infectious Diseases, National Institutes of Health, Bethesda, MD, 20892, USA; 4 Department of Physics, The George Washington University, D.C., 20052, USA; 5 Systems Biology Center, National Heart, Lung and Blood Institute, National Institutes of Health, Bethesda, MD 20892, USA.

## Abstract

Even though T-cell receptor (TCR) stimulation together with co-stimulation is sufficient for the activation of both naïve and memory T cells, the memory cells are capable of producing lineage specific cytokines much more rapidly than the naïve cells. The mechanisms behind this rapid recall response of the memory cells are still not completely understood. Here, we performed epigenetic profiling of human resting naïve, central and effector memory T cells using ChIP-Seq and found that unlike the naïve cells, the regulatory elements of the cytokine genes in the memory T cells are marked by activating histone modifications even in the resting state. Therefore, the ability to induce expression of rapid recall genes upon activation is associated with the deposition of positive histone modifications during memory T cell differentiation. We propose a model of T cell memory, in which immunological memory state is encoded epigenetically, through poising and transcriptional memory.

Long-lived memory T cells form the basis of adaptive immunity by orchestrating memory immune response. During secondary exposure, the response of memory T cells is both quantitatively and qualitatively different from the response of naïve T cells during initial exposure to pathogen^1^. Firstly, the number of T cells with specificity for the pathogen is higher due to clonal expansion of naïve precursors following primary exposure. In addition, antigen-exposed memory T cells are *qualitatively* different from naïve cells. Naïve T cells predominantly produce IL-2 as an initial response to activation. Only after several days of activation and polarization they can produce lineage-characteristic cytokines such as IFN-γ, IL-4, IL-5, IL-13 or IL-17. On the other hand, memory T cells are capable of producing these cytokines immediately^2^. This rapid recall ability, which allows the organism to fight pathogens faster and to limit the spread of infection, is the basis for vaccinations against numerous diseases^3^.

The molecular basis of the rapid recall response is still not well understood. In general, it is believed that both naïve and memory T cells receive the same signals from the antigen-presenting cells. Stimulation of TCR by MHC-antigen complex and co-stimulation, typically via B7-CD28 interaction, are sufficient for activation of both cell types. If anything, activation of memory cells may be less dependent on co-stimulation, although this point is still debated^4^. However, the same activation signaling leads to the induction of additional cytokine genes in memory T cells compared to naïve cells. Initial studies focused on identifying differences in signal transduction between the two cell types. In mouse CD4 T cells, initial TCR signaling works similarly in naïve and memory cells, but a key kinase, ZAP-70, is less phosphorylated in memory T cells compared to naïve T cells^5^, suggesting that memory T cells actually receive a weaker signal from TCR. In CD8 cells though, the initial TCR activation is similar and no difference in ZAP-70 phosphorylation was found^6^. Instead, it was reported that increased LAT concentration and phosphorylation in memory T cells led to increases in ERK and Jun phosphorylation upon activation^6^. MicroRNAs also were shown to play a role by regulating the expression of phosphatases that inhibit TCR signaling^7^. Another study reported that an important accessory molecule, SLP-76, is less phosphorylated in memory T cells than in naïve cells, again suggesting weaker TCR signaling in memory T cells^8^. Recent evidence in CD8 cells shows that the threshold for activation may indeed be higher in memory than in naïve cells^9^. However, despite these slight alterations in the levels of signaling molecules, the activation signals do reach transcription factors such as NF-κB, NFAT and AP1 in both naïve and memory T cells. As reported by Lai *et al*.^10^ in a murine model of immunity against influenza, NF-κB is activated and translocates to the nucleus at similar levels in both naïve and memory T cells but is only able to bind DNA and induce expression of *Ifng* in memory T cells. These findings suggest that rapid recall ability is mediated by the ability of transcription factors to bind to DNA at the appropriate genes, which is in turn regulated epigenetically by the local chromatin state.

Recently, we and other groups have profiled the epigenome of several immune cell populations. While the earlier studies established general relationship between chromatin modifications and gene expression in various cell types^11^,^12^,^13^, the later studies focused on unravelling the regulatory networks involved in the immune cell function^14^. These studies identified key regulatory elements and transcription factors that are involved in lineage specification during immediate Th1 and Th2 differentiaion^15^,^16^,^17^ in mice and humans and in human susceptibility to asthma^18^. Additionally, chromatin profiling was used to establish molecular basis for lineage plasticity in T helper cells^15^ and lineage relationship between several populations of cytotoxic T cells in mice^19^. Our previous studies have demonstrated that many inducible genes do not change their chromatin state during short-term T cell activation in activated CD4 T cells^20^. In fact, the majority of the activation-inducible genes already possessed positive chromatin modifications in the resting cells, in which these genes are silent. Therefore, we categorized those genes that are marked by activating histone modifications but not expressed as “poised”. It is possible that gene poising might be a manifestation of transcriptional memory acquired due to previous expression of the gene in the course of the natural history of the cell and retained in the cellular epigenome. On the basis of our results, we reasoned that gene poising might allow for rapid induction of the poised genes upon stimulus^20^. In the resting CD4 T cells, approximately 20% of all silent genes were poised by chromatin modifications and about 10% of these poised genes were subsequently induced upon T cell activation^20^. Among the genes poised in the resting T cells, we found a number of genes involved in cell cycle or metabolism. Most notably, cytokine genes including *IFNG* and *IL4* were poised. However, our studies with naïve murine T cells^15^ showed no poising at *Ifng* or *Il4* loci. These results suggest that cytokine genes are poised only in memory T cells, which may be related to their transcriptional history and persistent active epigenetic marks. This led us to hypothesize that cytokine gene poising imposed during T cell polarization underlies the rapid recall ability of memory T cells^21^.

In this study, we tested this hypothesis by profiling chromatin in three subsets of human CD4 T cells: naïve, central memory (TCM) and effector memory (TEM). We found that the ability of these subsets to express cytokines is associated with gene poising as indicated by the presence of positive chromatin modifications at promoters and enhancers of these genes. In general, the appearance of positive chromatin marks at the promoters and/or enhancers of genes upon differentiation of naïve T cells to TCM or TEM may lead not only to increased expression level of the genes in resting cells but also to increased inducibility of these genes upon activation, whereas loss of positive marks correlated with the loss of inducibility. These results support the hypothesis that the rapid recall ability of memory T cells is encoded in their epigenome.

## Results

**Figure 1:**
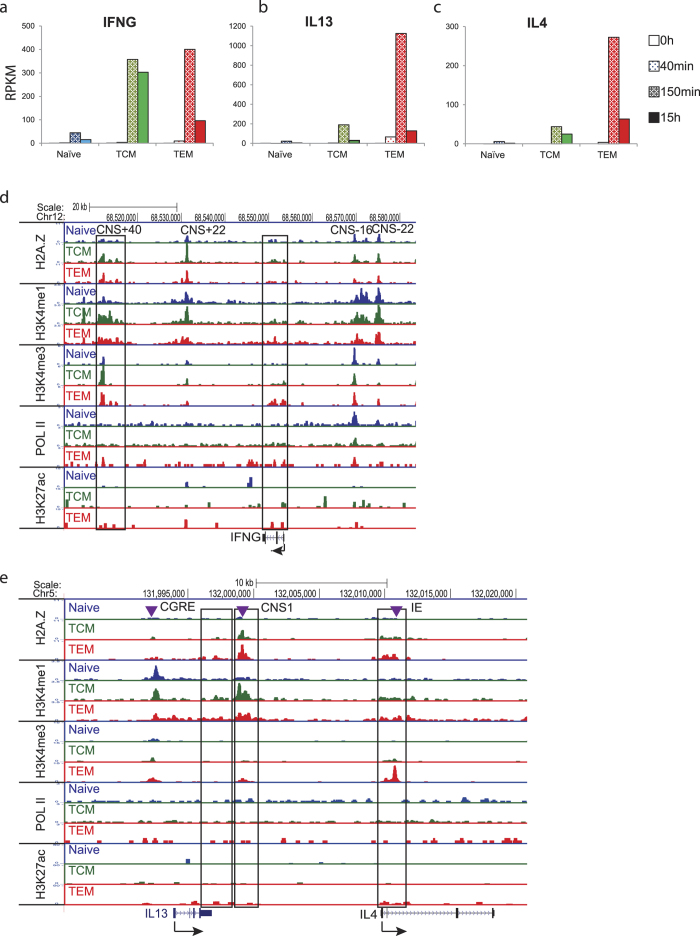
Enhancers and promoters of the cytokine genes are poised by active chromatin marks in TCM and TEM cells. Human resting naïve, TCM and TEM cells were isolated and activated with anti-CD3 and anti-CD28 beads for 40 min, 150 min or 15 hrs. Expression of the cytokine genes, *IFNG* (**a**), *IL13* (**b**) and *IL4* (**c**) was measured by RNA-Seq. Active chromatin marks (H3K4me1, H3K4me3, H3K27ac and H2A.Z) and RNA Polymerase II were profiled using ChIP-Seq in resting cells (0 hours). The Genome Browser tracks of the ChIP-Seq signals for the *IFNG* (**d**), and *IL4/13* (**e**) loci are shown. Y-axis shows coverage by estimated fragments normalized to millions of reads mapped (FPM). Putative regulatory elements are denoted on top and those areas that undergo epigenetic changes were framed. Purple arrows show the locations of combined *GATA3/MYB* elements as described previously^34^.

**Figure 2:**
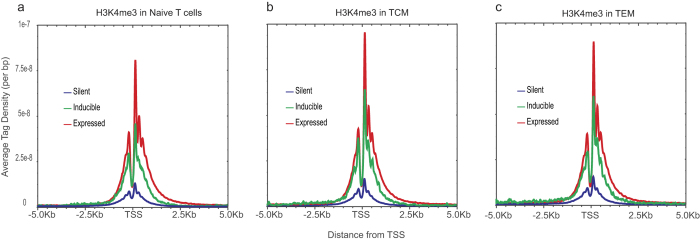
Inducible genes are poised. Average H3K4me3 tag density profiles were generated for genes that are expressed in resting cells, those that are silent in resting cells and those that are silent in resting cells but become induced upon activation. TSS, transcription start site.

Using cell surface markers, we separated total human CD4 T cells into 3 subsets (Fig. S1) that were reported to have differential ability to produce characteristic cytokines: (1) Naïve T cells (CD4^+^CD45RO^-^CD27^+^); (2) TCM (CD4^+^CD45RO^+^CD27^+^); and (3) TEM (CD4^+^CD45RO^+^CD27^−^). Naïve T cells were previously shown to be unable to produce IFN-γ or IL-4, whereas a minority of TCM and the majority of TEM cells were able to produce one of these cytokines upon activation^22^. TCM and TEM populations included cells of various lineages. Therefore, only a minority of the cells in a population could produce a particular cytokine (e.g. IFN-γ or IL-4)^22^. To examine whether the differential induction of cytokine genes between the CD4 T cell subsets is related to their epigenetic landscapes, we used ChIP-Seq to analyze genome-wide distribution of four positive chromatin marks (post-translational histone modifications H3K27ac, H3K4me1 and H3K4me3, histone variant H2A.Z) and RNA Polymerase II (Figs 1 and 2 and S2 and S3). We also measured gene expression in resting and activated cells using polyA RNA-Seq. Key ChIP-Seq experiments were replicated (see Methods).

RNA-Seq analysis revealed that neither naïve nor memory T cells expressed cytokine genes such as IFN-γ, IL-4, IL-13 or IL-17 in the resting state (Figs 1a,b,c and S3). Upon activation with anti-CD3/28 beads for 40 min, 150 min or 15 hrs, both TCM and TEM cells were able to produce much higher amounts of these cytokines than the naïve T cells (Figs 1a,b,c and S3). These rapid changes in mRNA abundance likely reflect changes in gene transcription, although post-transcriptional regulation cannot be excluded. Gene expression analysis at various time points post activation further showed that the expression of cytokine genes was more strongly and rapidly induced in memory T cells than in naïve T cells.

### The promoter and several enhancers of the IFNg gene are poised by active chromatin marks

Transcriptional regulation of *IFNG* locus has been extensively studied by multiple groups (reviewed in ref. 23). It has been reported that Th1 polarization leads to *IFNG* production, appearance of novel DNase I hypersensitive sites and increases in histone acetylation and other positive modifications in the vicinity of *IFNG* gene in murine and human T cells^15^,^23^,^24^,^25^,^26^,^27^,^28^. We examined whether the differences in histone modification profiles at *IFNG* locus in resting cells could explain the observed differential response to activation between naïve and memory T cells. Indeed, while some sites (e.g. CNS−16, CNS−22, CNS+22; Figs 1d and S2a) are modified similarly in both naïve and memory T cells, we observed appearance of positive histone marks at the *IFNG* Transcriptional Start Site (TSS), putative internal enhancer and CNS+40 (Fig. 1d, outlined) in memory cells. In addition, Pol II was not present at the *IFNG* promoter in the resting cells (Fig. 1d). Presence of these marks in memory T cells correlated with IFNG inducibility. With the exception of the internal enhancer/insulator that is bound by CTCF and is involved in long-distance chromatin interactions within the *IFNG* locus (Fig. S2a and ref. 29), the functional significance of the differentially modified sites in human T cells is not fully understood. However, in murine T cells, many of the homologous sites were shown to bind TBX-21/T-BET, NFκB, STAT-4 and other transcription factors^30^, suggesting that they are functional regulatory elements in humans as well.

Interestingly, we observed relatively low ChIP-Seq enrichment peaks at the IFNG promoter (e.g. compared to housekeeping genes, Fig. S3q–s) even in memory T cells, which suggests that this locus is modified in only a fraction of the cells. This likely reflects the fact that only a fraction of TEM cells belong to Th1 lineage and are capable of producing IFNG^22^.

Presence of positive chromatin marks at some of the enhancers in the naïve T cells (Fig. 1d) suggests that locus opening happens in a stepwise manner and that multiple events, which occur during early development of T cells, are required to confer full inducibility in differentiated Th1 cells. Taking this into account, we decided to analyze developmental history of T cells on the basis of our own^31^ and ENCODE^32^ data. Indeed, the *IFNG* locus (Fig. S2a) has no positive marks in H7 embryonic stem cells (ESC). CD34 hematopoietic stem cells (HSC) have gained low levels of H2A.Z at the distal elements (+118 kb and −64 kb). Data for common myeloid and lymphoid progenitors (CMP and CLP, respectively) are not available, but CD36 proerythroblasts and CD14 monocytes do not gain further modifications at this locus (data not shown). However, fellow lymphocyte CD20 B cells seem to have acquired positive modifications at CNS-16 and CNS-22 elements (Fig. S2a), suggesting that the *IFNG* locus may have begun to open at the CLP stage. The sites that are positively modified early in lymphocyte development (e.g. CNS-22^26^) could potentially serve as Locus Control Regions (LCRs). Interestingly, during Th1/2 polarization of naïve T cells, strong deposition of H3K4me1 can be already observed at 72 h post activation, whereas H3K4me3 deposition likely requires longer time and/or additional stimuli^16^.

### Several enhancers of the Th2 cytokine genes are poised by active chromatin marks

The Th2 cytokine locus contains *Il4, Il5* and *Il13* genes, as well as *RAD50* and *KIF3A* (reviewed in ref. 33 and Figs 1e and S2b). Unlike *RAD50* and *KIF3A*, which are weakly expressed in all three subsets (not shown), the cytokine genes are exclusively expressed in activated memory T cells (Fig. 1b,c). The ability of memory T cells to induce *IL4* and *IL13* expression correlates with the appearance of positive histone marks at the loci known as CNS1 and IE (Fig. 1e, boxed). Interestingly, these sites have been shown to be enhancers that bind GATA-3 and MYB transcription factors^34^. While GATA-3 is universally expressed in T cells, MYB is expressed at low levels in resting naïve T cells, and its expression is induced late during Th2 polarization. MYB has been shown to interact with MLL proteins and may be responsible for the deposition of positive marks at the enhancers in the Th2 locus^34^,^35^.

We observed that several peaks of H2A.Z and H3K4me1/3 (in particular, those at the *RAD50* and *KIF3A* promoters) are already present at this genomic locus in naïve T cells (Fig. S2b and data not shown), suggesting that the Th2 locus is partially primed for expression during T cell development. Stepwise deposition of histone modifications starts in ESCs with the deposition of low levels of H3K4me3 at CGRE (Fig. S2b) and continues with the deposition of positive marks at CNS1 and HSV elements in CD34 HSCs. B cells have low levels of H2A.Z and H3K4me3 at CGRE and CNS1. The modifications at the IE site are observed only in the memory T cells and correlate with *IL4* and *IL13* inducibility.

### Many other cytokines are also poised

Next, we examined the histone modifications at several other cytokine genes, including *IL17A, IL3, IL9* and *IL22* (Fig. S3), which are not expressed in resting cells and are more inducible in memory T cells than in naïve T cells. This analysis revealed new peaks of activating histone modifications in the memory T cells, consistent with the inducibility of these genes in the memory cells. This finding is in contrast to another T-cell cytokine CCL5 (RANTES), whose accelerated production by memory T cells was previously reported to be regulated post transcriptionally^36^. Our results confirm that *CCL5* is expressed at a higher level in memory than in naïve T cells even in the resting state. While the increase in basal expression does correlate with the appearance of positive peaks at the promoter, transcriptional induction upon activation was not observed either in memory or in naïve cells (data not shown).

### Epigenetic environment at master regulator genes

Transcription factors TBX-21 (T-BET), GATA-3 and RORC are considered to be the master regulators of the Th1, Th2 and Th17 lineages, respectively^37^,^38^,^39^. *TBX21* is not expressed in naïve T cells and is induced up to 100-fold upon activation. Memory cells express low levels of *TBX21* mRNA, and it is strongly induced upon activation (Fig. S3g,i). As could be expected, we observed a substantial increase in the H3K4me3 level at the *TBX21* promoter, which correlated with increased and more rapid *TBX21* induction in the memory cells. The TH17 transcription factor *RORC* locus is silent in resting naïve T cells but is expressed and inducible in memory T cells (Fig. S3b,c,e). This correlates with the appearance of H3K4me3 islands at both of its promoters even though only the shorter transcript was detected by RNA-Seq. Finally, the Th2 transcription factor GATA-3 is expressed in both naïve and memory T cells, although at a higher level in memory T cells (Fig. S3f,h). We observed no chromatin changes at the *GATA3* locus.

### Genome-wide epigenetic changes correlate with inducibility

Our data suggest that many of the inducible cytokines and other genes are differentially epigenetically modified at promoter and/or enhancer elements between naïve and memory T cells. To examine whether other inducible genes are also poised, we generated average tag density profiles for three groups of genes: (1) expressed in resting T cells; (2) silent in resting T cells but induced upon activation; and (3) silent in both resting and activated T cells (Figs 2 and S4). Similar to our previous results^20^, we found that the inducible genes have higher levels of positive marks at their promoters than silent genes and are poised for induction.

**Figure 3:**
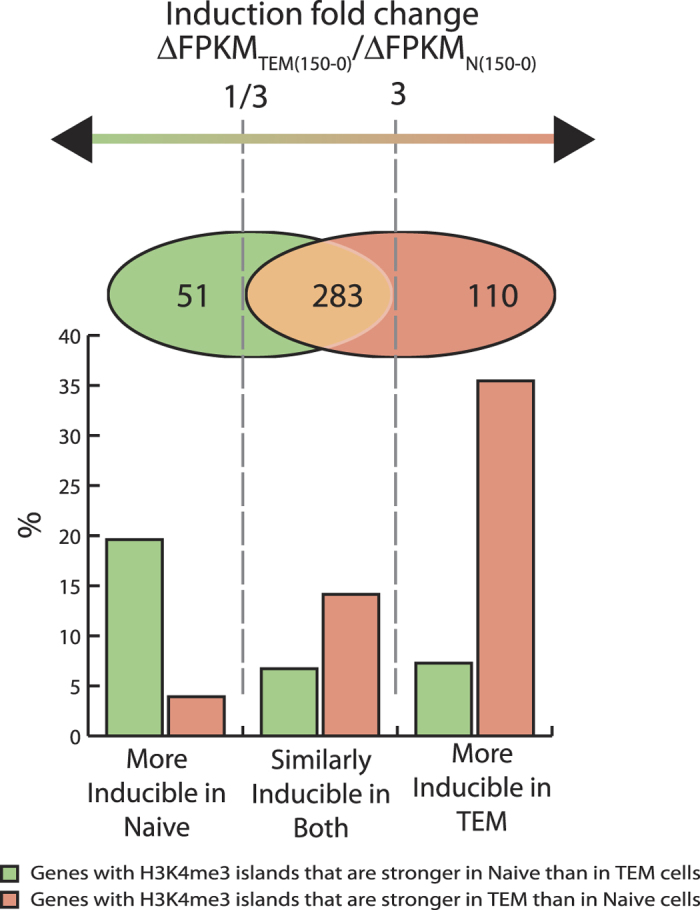
Differential gene inducibility can be explained by chromatin state. Genes that were induced upon activation in either naive or effector memory T cells were categorized into 3 groups: more inducible in Naïve, more inducible in TEM or inducible in both (as shown by Venn diagram). Bar graphs show the percentage of genes in each category that have gained or lost H3K4me3 in their vicinity [−20 kb from TSS, +1 kb from TSS] during transition from naïve to memory state.

In order to determine whether differential modifications at the enhancers and promoters could explain the *differential* gene induction between naïve and memory T cells, we identified islands of histone modifications in the vicinity of genes that are differentially induced between the subsets. We found that for ~35% of the genes that are strongly induced in TEM but not in naïve T cells, the gene induction was associated with the appearance of positive H3K4me3 marks in the vicinity of the gene in the course of differentiation of naïve T cells into memory T cells (Figs 3 and S5).

### Deposition of positive marks leads to increased expression or inducibility

**Figure 4:**
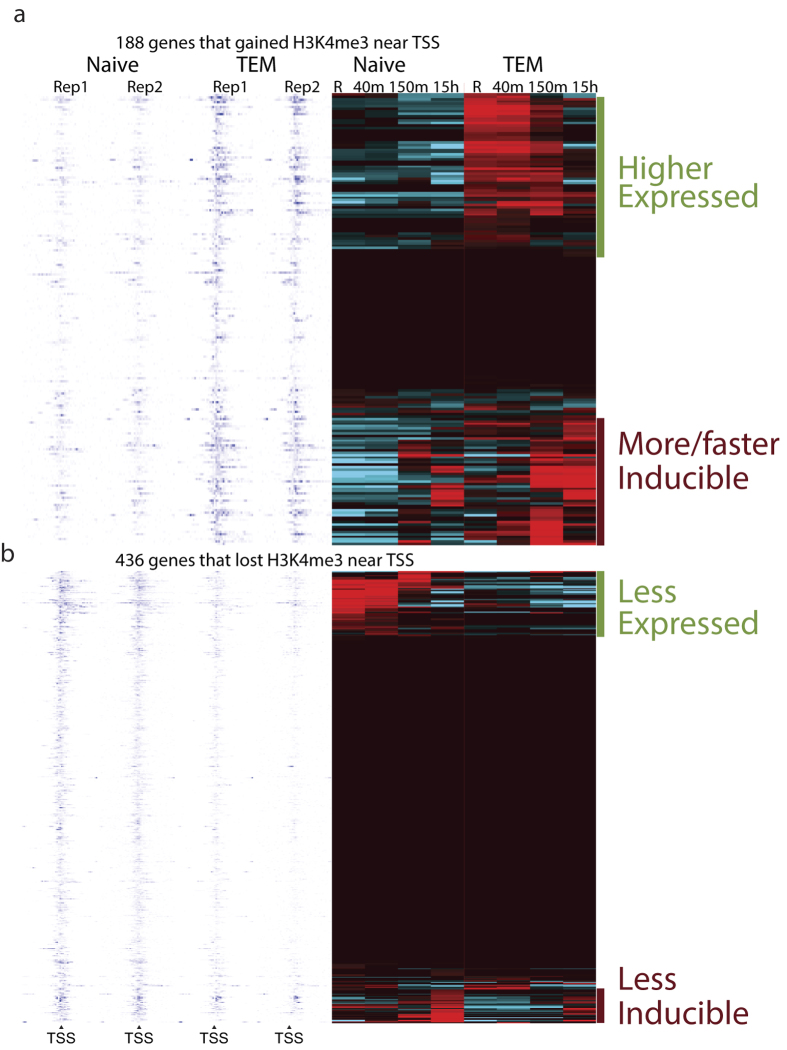
Gain of H3K4me3 at promoter may lead to increased expression and/or inducibility, whereas loss of H3K4me3 may lead to loss of expression and/or inducibility. On the left tag density heatmaps show the level of H3K4me3 within 5 kb around TSS for genes that gained (**a**) or lost (**b**) H3K4me3 islands at the promoter in the transition from naïve T cells (Naïve) to effector memory T cells (TEM). On the right heatmaps show level of expression for the corresponding genes in resting cells (R) and cells activated for the period shown.

In order to evaluate the transcriptional consequences of gain or loss of activating histone modifications, we systematically analyzed the expression levels of genes that gain or lose the positive marks near their promoters during their transition from naïve to memory T cells. For this, we identified genes with differentially modified promoters: 112 gene promoters became H3K4me3 modified upon transition of T cells from naïve to TCM and 188 from naïve to TEM (Figs 4 and S6). Interestingly, only about half of the genes that gained positive modifications at their promoters displayed increased expression or inducibility. The rest of the genes that gained modifications did not change their expression state and stayed silent and uninducible. It is not clear whether these genes were expressed during transition from naïve to memory cells. It is possible that a stimulus other than activation is needed to induce their expression in memory T cells. A majority of the genes that lost H3K4me3 in the transition from naïve to memory state, were not expressed even in naïve T cells. Those genes that were either expressed or inducible in naïve T cells lost that inducibility. On the basis of gene ontology analysis, the set of genes that gained H3K4me3 in the transition from naïve T cell to TEM was highly enriched in GO terms related to immune response, chemotaxis and cell adhesion (Table S1), whereas the gene set that lost modifications was much more diverse (as shown by less significant p values). The gene set that lost modifications upon transition from naïve to memory state was enriched for terms related to neuronal, vascular and organ development, as well as cell adhesion and motility. Presence of “cell adhesion” on both lists likely reflects differential trafficking of these cell types.

## Discussion

Although there has been substantial progress in the understanding of the development of populations of memory T cells, we comprehend relatively little about the mechanisms responsible for their increased functionality. In this study, we demonstrate that the cytokine genes integral to T cell function and protective immunity are “poised” and ready for rapid induction in memory T cells. It is likely that poising is a critical aspect of protective immunity as it allows the host to have a leg up on pathogens previously encountered, which would be evolutionarily advantageous for the host. Thus, although it is clear that memory T cells are increased in number during a secondary challenge, their increased ability to produce cytokines likely contributes to protection against repeat infections with the same pathogen.

**Figure 5:**
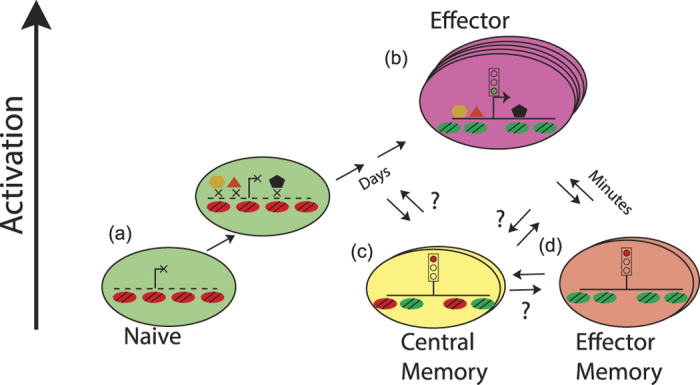
Model: Epigenetic poising of cytokine genes. (**a**) In naïve T cells, genes required for effector function exist in closed chromatin environment (red nucleosomes). Even though transcription factors such as NFκB are activated, they are unable to bind to their targets due to the closed chromatin state. Upon activation, naïve T cells proliferate and in the course of several days remodel their chromatin. This allows for transcription factor binding, recruitment of RNA Polymerase II and expression of cytokines (**b**). After elimination of infection, surviving effector T cells become memory T cells (**c** and **d**). These cells do not express effector genes, but positive chromatin modifications (green nucleosomes) remain at promoters and/or enhancers and maintain the genes in a poised state, allowing immediate transcription factor binding and rapid expression of effector genes upon repeat activation. The exact differentiation pathway of central memory T cells (**c**) is not known. The opening of effector gene chromatin in these cells is incomplete compared to TEM cells. Based on ref. 21.

Several recent studies have profiled epigenomes of human and mouse T cell populations during rest^11^,^12^,^13^,^19^ or upon T cell polarization^15^,^16^,^17^,^40^. These studies have correlated the presence of chromatin modifications with the level of ongoing gene expression^11^,^12^,^13^, described polarization^16^,^17^,^41^ or disease state-related^18^ regulatory elements. However, whether and how histone modification marks that exist in the resting naïve and memory cells influence future gene inducibility during T cell activation remains unknown. Our study provides evidence suggesting that the rapid recall ability of memory T cells is determined by the epigenetic status of rapid recall genes in the resting state. In particular, in memory T cells, we observed the appearance of islands of positive histone modifications at promoters and enhancer elements of *IFNG, IL4, IL9, IL13, IL17A* and other genes that are strongly upregulated during secondary immune response. Upon differentiation of naïve T cells into effectors following primary activation, these characteristic cytokine genes were expressed. Upon cessation of immune response and transition of effectors into memory T cells, these cytokine genes were no longer expressed, but their promoter and enhancer elements remained positively marked, thus maintaining the genes’ chromatin environment and poised state (Fig. 5). This poised state in turn resulted in faster induction of these genes upon secondary stimulus, likely due to the chromatin environment being conducive to sequence-specific and general transcription factor binding and gene expression. Recently, Bevington *et al*. uncovered the presence of stably maintained DNase-hypersensitive sites in the murine memory T cells^42^ which further supports our findings. Here we report that in human memory T cells positive chromatin modifications are also present in the vicinity of rapid recall genes. Interestingly, not all genes that gained positive marks during Naïve to memory transition become inducible during T cell activation (Fig. 4). It is possible, that additional chromatin changes (e.g. demethylation) may be required. Alternatively, these genes may need signals other than short term aCD3/aCD28 stimulation for induction.

Our results also provide insights into relationship between central and effector memory Th cells. Although central memory cells can produce cytokines more efficiently than naïve cells, they produce less cytokines than the activated effector memory cells. This correlates with a “less poised” state of their chromatin. This suggests that these cells have branched off T cell differentiation pathway earlier than Tem cells, although the exact relationship between these populations remains unclear.

In this study, we examined the total TCM and TEM cells. These populations include T cells that belong to different lineages, including Th1, Th2 and Th17. Therefore, we observed positive modifications at cytokine genes specific for all three of these lineages. We believe that individual cells will have open chromatin structure only at some of these loci in accordance with their lineages, but further work is needed to provide experimental evidence for this. Because of this heterogeneity and our focus on rapid recall gene induction, we had to limit our analysis to “positive” marks: gain of signal is easy to detect even when the signal is present only in a small fraction of population, whereas a much more homogenous population is needed to detect the expected loss of “negative” marks.

Since histone-modifying enzymes can be recruited by transcription factors, RNA Polymerase II itself, other histone modifications and RNA^43^, we tried to identify transcription factor binding sites that might be responsible for the deposition of histone modifications during differentiation from naïve T cells to TEM. We used Clover software^44^ to compare enrichment of transcription factor binding sites (TFBS) in peaks exclusively present in TEM compared to that of those peaks that are present in naïve cells. We found that 260 TFBS matrices from the Transfac database showed significant enrichment in this comparison (Table S2), whereas 869 showed significant depletion. Among the enriched TFBS we found those for transcription factors such as IRFs, RUNXs, ETS family, MAF and others. Many of these factors, including IRF1, IRF4, IRF8, JUN, JUNB, JUND, FOS, FOSL1, MYC and TFAP were inducible during activation of naïve T cells, whereas others (e.g. some of ETSs and RUNXs) were expressed constitutively and may be responsible for the maintenance of positive modifications in these areas after activating signal is gone. RUNX1 and ETS1 were indeed reported to bind areas of open chromatin that remain accessible in resting memory cells^42^. Although TBX21 and RORC were not in the Clover database, the binding sites for their close homologues TBX5, RORA and RORB^45^,^46^ were enriched. A matrix created using the TBX21 binding sites from Szabo *et al*.^37^ was also significantly enriched in the TEM-specific H3K4me3 islands. Interestingly, the factors such as NFAT, NFκB or EGR were either not enriched or significantly depleted, suggesting that these factors do not serve as pioneer factors that create open chromatin but rather bind the sites of open chromatin structure.

In summary, we have shown that many of the genes that are differentially inducible between naïve and memory T cells reside in a different chromatin environment in memory compared to naïve T cells. Appearance of open chromatin marks at the promoters and/or enhancer elements correlates with increased expression and/or inducibility. In particular, this relates to important cytokine genes such as *IFNG, IL4, IL9, IL13* and others. These findings suggest that the rapid recall response of memory T cells is mediated, at least partially, by the poised chromatin environment that exists in the memory T cells around these genes.

## Methods

### Cells

T cells were purified from lymphopaks obtained from NIH Blood Center or blood filters obtained from University of Cincinnati Hoxworth Blood Center. Cells from 2–4 healthy donors were combined for each experiment. Human CD4 T cells were enriched by negative selection using Invitrogen or Miltenyi Untouched CD4 T cell Purification Kit. Cells were further sorted into populations, as shown in Fig. S1, using antibodies against CD4 (RPA-T4), CD45RO (UCHL1) and CD27 (L128A) and DAPI.

### ChIP-Seq

For ChIP, chromatin was prepared by formaldehyde cross-linking and sonication. ChIP-Seq was performed as described previously^11^ (see refs 47 and 48 also for review and protocol, respectively) either manually or using SX-8G robot (Diagenode). Key ChIP-Seq experiments were repeated using a different antibody (H3K4me3) or an alternative lot of the same polyclonal antibody (H3K4me1 and H2A.z) and T cells obtained from different donors. Replicates yielded similar results (Fig. S7). Antibodies used for ChIP were Abcam ab8895 (H3K4me1), ab8580 or Cell Signaling CS200580 (H3K4me3), ab4174 (H2A.Z), Active Motif 4H8 (Pol II) and Diagenode pAb-196–060 (H3K27ac). Antibody specificity was assessed by the manufacturers and/or in a previous study^12^. Sequencing for 36–75 bases was performed using Illumina GAII/x/HiSeq2500. Data analysis was conducted in BioWardrobe^49^. Briefly, ChIP-Seq data were aligned by Bowtie to the human genome (hg19); only unique reads with no more than 1 mismatch were kept. Reads were extended to estimated fragment length, normalized to total mapped read number and displayed as coverage on a mirror of the University of California Santa Cruz (UCSC) genome browser. MACS2^50^ was used to identify islands of enrichment. Data have been deposited to GEO under GSE89404 accession number.

### RNA-Seq

For RNA-Seq, activation was performed by addition of anti-CD3/28 beads (Invitrogen) to cells for 40 min, 150 min or 15 hrs according to the manufacturer’s instructions. mRNA-Seq libraries were constructed using Illumina TruSeq kit. Data analysis was performed in BioWardrobe^49^. Briefly, RNA-Seq data were aligned by STAR^51^ to the human genome (hg19) with refsec annotation taken from the UCSC Genome Browser and RPKM values for isoforms were estimated in BioWardrobe. For isoforms with the same TSS, RPKM scores were combined.

**Data Analysis:** Figs 2 and S4. Average read density profiles were calculated around transcription start sites for the three gene sets using BioWardrobe^49^. “Expressed” genes had FPKM > 5 in resting cells, whereas “Silent” genes had FPKM <2. “Inducible” genes had FPKM <2 in resting cells and >5 after activation. According to observations from^52^ and our own experience with spike-in controls (not shown) one copy per cell corresponds to ~5–8 RPKM. While we cannot entirely exclude the possibility that the genes under 2 RPKM are expressed at a very low level: e.g. due leaky Pol II pausing, we believe that expression of the genes we consider silent here (<2 RPKM) is below one copy per cell. To create average density plots, reads within 2000 bases upstream and downstream from start sites were shifted by ½ average fragment length in the direction of each read, counted at each position and normalized by the number of total mapped reads and number of genes in each subset. Reads belonging to the X and Y chromosomes were counted twice. Resulting graphs were smoothed with the window of 100.

**Data Analysis:** Figs 3 and S5. Genes that were silent in resting T cells (<2 FPKM) and induced to more than 5 FPKM after 150 min activation in either Naïve or TEM cells (Naïve or TCM for Fig. S5) were ranked by their relative induction ((ABS (FPKM_TEM 150_ − FPKM_TEM R_) + 0.1)/(ABS (FPKM_Naïve 150_ − FPKM_Naïve R_) + 0.1) and separated into three groups: those that are at least 3 fold more inducible in Naïve; at least 3 fold more inducible in TEM; similarly inducible (As shown on Venn diagram). The percentage of genes that had significantly (MAnorm^53^ p-value < 0.01, adjusted fold change >2, 15 reads in at least one condition) stronger islands in one cell type but not another within [TSS−20 kb; TSS + 1 kb] interval are shown by bar graphs.

**Data Analysis:** Figs 4 and S6. (Heatmaps) Genes that had significantly different level of H3K4me3 at their promoters (TSS+/−1 kb, MAnorm^53^ p-value < 0.01, adjusted fold change >2 in both replicates) in memory vs. naive cells were selected for analysis. To avoid huge changes in very low expressed genes, RPKM values below 1 were increased to 1. Clustering of expression values was performed using AltAnalyze^54^ and HOPACH^55^ algorithm; tag density heatmaps were made in BioWardrobe^49^.

## Additional Information

**How to cite this article:** Barski, A. *et al*. Rapid Recall Ability of Memory T cells is Encoded in their Epigenome. *Sci. Rep.* **7**, 39785; doi: 10.1038/srep39785 (2017).

**Publisher's note:** Springer Nature remains neutral with regard to jurisdictional claims in published maps and institutional affiliations.

## Supplementary Material

Saupplementary Figures and Table 1

Saupplementary Table 2

## References

[b1] Sprent J. & Surh C. D. T cell memory. Annu. Rev. Immunol. 20, 551–79 (2002). 11861612 10.1146/annurev.immunol.20.100101.151926

[b2] Farber D. L. Biochemical signaling pathways for memory T cell recall. Semin. Immunol. 21, 84–91 (2009). 19298946 10.1016/j.smim.2009.02.003 PMC2683752

[b3] Kaech S. M., Wherry E. J. & Ahmed R. Effector and memory T-cell differentiation: implications for vaccine development. Nat. Rev. Immunol. 2, 251–62 (2002). 12001996 10.1038/nri778

[b4] Croft M., Bradley L. M. & Swain S. L. Naive versus memory CD4 T cell response to antigen. Memory cells are less dependent on accessory cell costimulation and can respond to many antigen-presenting cell types including resting B cells. J. Immunol. 152, 2675–85 (1994). 7908301

[b5] Farber D. L., Acuto O. & Bottomly K. Differential T cell receptor-mediated signaling in naive and memory CD4 T cells. Eur. J. Immunol. 27, 2094–101 (1997). 9295050 10.1002/eji.1830270838

[b6] Kersh E. N. . TCR signal transduction in antigen-specific memory CD8 T cells. J. Immunol. 170, 5455–63 (2003). 12759421 10.4049/jimmunol.170.11.5455

[b7] Li Q. J. . miR-181a Is an Intrinsic Modulator of T Cell Sensitivity and Selection. Cell 129, 147–161 (2007). 17382377 10.1016/j.cell.2007.03.008

[b8] Hussain S. F., Anderson C. F. & Farber D. L. Differential SLP-76 expression and TCR-mediated signaling in effector and memory CD4 T cells. J. Immunol. 168, 1557–65 (2002). 11823482 10.4049/jimmunol.168.4.1557

[b9] Mehlhop-Williams E. R. & Bevan M. J. Memory CD8+ T cells exhibit increased antigen threshold requirements for recall proliferation. J. Exp. Med. 211, 345–56 (2014). 24493801 10.1084/jem.20131271 PMC3920562

[b10] Lai W. . Transcriptional control of rapid recall by memory CD4 T cells. J. Immunol. 187, 133–40 (2011). 21642544 10.4049/jimmunol.1002742 PMC3131107

[b11] Barski A. . High-resolution profiling of histone methylations in the human genome. Cell 129, 823–37 (2007). 17512414 10.1016/j.cell.2007.05.009

[b12] Wang Z. . Combinatorial patterns of histone acetylations and methylations in the human genome. Nat. Genet. 40, 897–903 (2008). 18552846 10.1038/ng.154 PMC2769248

[b13] Araki Y. . Genome-wide Analysis of Histone Methylation Reveals Chromatin State-Based Regulation of Gene Transcription and Function of Memory CD8+ T Cells. Immunity 30, 912–925 (2009). 19523850 10.1016/j.immuni.2009.05.006 PMC2709841

[b14] Winter D. R., Jung S. & Amit I. Making the case for chromatin profiling: a new tool to investigate the immune-regulatory landscape. Nat. Rev. Immunol. 15, 585–94 (2015). 26272294 10.1038/nri3884

[b15] Wei G. . Global mapping of H3K4me3 and H3K27me3 reveals specificity and plasticity in lineage fate determination of differentiating CD4+ T cells. Immunity 30, 155–67 (2009). 19144320 10.1016/j.immuni.2008.12.009 PMC2722509

[b16] Hawkins R. D. . Global chromatin state analysis reveals lineage-specific enhancers during the initiation of human T helper 1 and T helper 2 cell polarization. Immunity 38, 1271–84 (2013). 23791644 10.1016/j.immuni.2013.05.011 PMC4607036

[b17] Vahedi G. . STATs shape the active enhancer landscape of T cell populations. Cell 151, 981–93 (2012). 23178119 10.1016/j.cell.2012.09.044 PMC3509201

[b18] Seumois G. . Epigenomic analysis of primary human T cells reveals enhancers associated with TH2 memory cell differentiation and asthma susceptibility. Nat. Immunol. 15, (2014). 10.1038/ni.2937 PMC4140783 24997565

[b19] Crompton J. G. . Lineage relationship of CD8(+) T cell subsets is revealed by progressive changes in the epigenetic landscape. Cell. Mol. Immunol. 13, 1–12 (2015). 10.1038/cmi.2015.32 PMC4947817 25914936

[b20] Barski A. . Chromatin poises miRNA- and protein-coding genes for expression. Genome Res. 19, 1742–51 (2009). 19713549 10.1101/gr.090951.109 PMC2765269

[b21] Cuddapah S., Barski A. & Zhao K. Epigenomics of T cell activation, differentiation, and memory. Curr. Opin. Immunol. 22, 341–7 (2010). 20226645 10.1016/j.coi.2010.02.007 PMC2892201

[b22] Okada R., Kondo T., Matsuki F., Takata H. & Takiguchi M. Phenotypic classification of human CD4+ T cell subsets and their differentiation. Int. Immunol. 20, 1189–99 (2008). 18635582 10.1093/intimm/dxn075

[b23] Lee G. R., Kim S. T., Spilianakis C. G. & Fields P. E. & Flavell, R. a. T helper cell differentiation: regulation by cis elements and epigenetics. Immunity 24, 369–79 (2006). 16618596 10.1016/j.immuni.2006.03.007

[b24] Chang S. & Aune T. M. Histone hyperacetylated domains across the Ifng gene region in natural killer cells and T cells. Proc. Natl. Acad. Sci. USA 102, 17095–100 (2005). 16286661 10.1073/pnas.0502129102 PMC1283154

[b25] Shnyreva M. . Evolutionarily conserved sequence elements that positively regulate IFN-gamma expression in T cells. Proc. Natl. Acad. Sci. USA 101, 12622–7 (2004). 15304658 10.1073/pnas.0400849101 PMC515107

[b26] Hatton R. D. . A distal conserved sequence element controls Ifng gene expression by T cells and NK cells. Immunity 25, 717–29 (2006). 17070076 10.1016/j.immuni.2006.09.007

[b27] Zhu J. & Paul W. E. CD4 T cells: fates, functions, and faults. Blood 112, 1557–69 (2008). 18725574 10.1182/blood-2008-05-078154 PMC2518872

[b28] Agarwal S. & Rao A. Modulation of chromatin structure regulates cytokine gene expression during T cell differentiation. Immunity 9, 765–775 (1998). 9881967 10.1016/s1074-7613(00)80642-1

[b29] Sekimata M. . CCCTC-binding factor and the transcription factor T-bet orchestrate T helper 1 cell-specific structure and function at the interferon-gamma locus. Immunity 31, 551–64 (2009). 19818655 10.1016/j.immuni.2009.08.021 PMC2810421

[b30] Balasubramani A. . Modular utilization of distal cis-regulatory elements controls Ifng gene expression in T cells activated by distinct stimuli. Immunity 33, 35–47 (2010). 20643337 10.1016/j.immuni.2010.07.004 PMC2994316

[b31] Cui K. . Chromatin signatures in multipotent human hematopoietic stem cells indicate the fate of bivalent genes during differentiation. Cell Stem Cell 4, 80–93 (2009). 19128795 10.1016/j.stem.2008.11.011 PMC2785912

[b32] The Encode Project Consortium. A user’s guide to the encyclopedia of DNA elements (ENCODE). PLoS Biol. 9, e1001046 (2011). 21526222 10.1371/journal.pbio.1001046 PMC3079585

[b33] Ansel K. M., Djuretic I., Tanasa B. & Rao A. Regulation of Th2 differentiation and Il4 locus accessibility. Annu. Rev. Immunol. 24, 607–56 (2006). 16551261 10.1146/annurev.immunol.23.021704.115821

[b34] Kozuka T., Sugita M., Shetzline S., Gewirtz A. M. & Nakata Y. c-Myb and GATA-3 cooperatively regulate IL-13 expression via conserved GATA-3 response element and recruit mixed lineage leukemia (MLL) for histone modification of the IL-13 locus. J. Immunol. 187, 5974–82 (2011). 22039304 10.4049/jimmunol.1100550 PMC3221810

[b35] Jin S. . c-Myb binds MLL through menin in human leukemia cells and is an important driver of MLL-associated leukemogenesis. J. Clin. Invest. 120, 593–606 (2010). 20093773 10.1172/JCI38030 PMC2810070

[b36] Swanson B. J., Murakami M., Mitchell T. C., Kappler J. & Marrack P. RANTES production by memory phenotype T cells is controlled by a posttranscriptional, TCR-dependent process. Immunity 17, 605–15 (2002). 12433367 10.1016/s1074-7613(02)00456-9

[b37] Szabo S. J. . A novel transcription factor, T-bet, directs Th1 lineage commitment. Cell 100, 655–69 (2000). 10761931 10.1016/s0092-8674(00)80702-3

[b38] Zheng W. & Flavell R. a. The transcription factor GATA-3 is necessary and sufficient for Th2 cytokine gene expression in CD4 T cells. Cell 89, 587–96 (1997). 9160750 10.1016/s0092-8674(00)80240-8

[b39] Ivanov I. I. . The orphan nuclear receptor RORgammat directs the differentiation program of proinflammatory IL-17+ T helper cells. Cell 126, 1121–33 (2006). 16990136 10.1016/j.cell.2006.07.035

[b40] Lu K. T. . Functional and Epigenetic Studies Reveal Multistep Differentiation and Plasticity of *In Vitro*-Generated and *In Vivo*-Derived Follicular T Helper Cells. Immunity 35, 622–632 (2011). 22018472 10.1016/j.immuni.2011.07.015 PMC3235706

[b41] Wei G. . Global mapping of H3K4me3 and H3K27me3 reveals specificity and plasticity in lineage fate determination of differentiating CD4+ T cells. Immunity 30, 155–67 (2009). 19144320 10.1016/j.immuni.2008.12.009 PMC2722509

[b42] Bevington S. L. . Inducible chromatin priming is associated with the establishment of immunological memory in T cells. EMBO J. 35, 515–535 (2016). 26796577 10.15252/embj.201592534 PMC4772849

[b43] Ruthenburg A. J., Allis C. D. & Wysocka J. Methylation of lysine 4 on histone H3: intricacy of writing and reading a single epigenetic mark. Mol. Cell 25, 15–30 (2007). 17218268 10.1016/j.molcel.2006.12.014

[b44] Frith M. C. . Detection of functional DNA motifs via statistical over-representation. Nucleic Acids Res. 32, 1372–81 (2004). 14988425 10.1093/nar/gkh299 PMC390287

[b45] Wilson V. & Conlon F. L. The T-box family. Genome Biol. 3, REVIEWS3008 (2002). 10.1186/gb-2002-3-6-reviews3008 PMC139375 12093383

[b46] Jetten A. M. Retinoid-related orphan receptors (RORs): critical roles in development, immunity, circadian rhythm, and cellular metabolism. Nucl. Recept. Signal. 7, e003 (2009). 19381306 10.1621/nrs.07003 PMC2670432

[b47] Barski A. & Zhao K. Genomic location analysis by ChIP-Seq. J. Cell. Biochem. 107, 11–8 (2009). 19173299 10.1002/jcb.22077 PMC3839059

[b48] Cuddapah S. . Native chromatin preparation and illumina/solexa library construction. Cold Spring Harb. Protoc. 4, 1–8 (2009). 10.1101/pdb.prot5237 PMC3541822 20147195

[b49] Kartashov A. V. & Barski A. BioWardrobe: an integrated platform for analysis of epigenomics and transcriptomics data. Genome Biol. 16, 158 (2015). 26248465 10.1186/s13059-015-0720-3 PMC4531538

[b50] Zhang Y. . Model-based analysis of ChIP-Seq (MACS). Genome Biol. 9, R137 (2008). 18798982 10.1186/gb-2008-9-9-r137 PMC2592715

[b51] Dobin A. . STAR: Ultrafast universal RNA-seq aligner. Bioinformatics 29, 15–21 (2013). 23104886 10.1093/bioinformatics/bts635 PMC3530905

[b52] Mortazavi A., Williams B. A., McCue K., Schaeffer L. & Wold B. Mapping and quantifying mammalian transcriptomes by RNA-Seq. Nat Methods 5, 621–628 (2008). 18516045 10.1038/nmeth.1226 PMC13303166

[b53] Shao Z., Zhang Y., Yuan G.-C., Orkin S. H. & Waxman D. J. MAnorm: a robust model for quantitative comparison of ChIP-Seq data sets. Genome Biol. 13, R16 (2012). 22424423 10.1186/gb-2012-13-3-r16 PMC3439967

[b54] Emig D. . AltAnalyze and DomainGraph: analyzing and visualizing exon expression data. Nucleic Acids Res. 38, W755–62 (2010). 20513647 10.1093/nar/gkq405 PMC2896198

[b55] van der Laan M. J. & Pollard K. S. A new algorithm for hybrid hierarchical clustering with visualization and the bootstrap. J. Stat. Plan. Inference 117, 275–303 (2003).

