# Supplementary material for: Rapid Recall Ability of Memory T cells is Encoded in their Epigenome
Source: Sci Rep. 2017 Jan 5;7:39785. doi: 10.1038/srep39785 (PMC5215294; doi:10.1038/srep39785)
Supplement: Saupplementary Figures and Table 1 [file srep39785-s1.pdf]

# Supplementary Information

## Rapid Recall Ability of Memory T cells is Encoded in Their Epigenome

Artem Barski[1*#], Suresh Cuddapah[2*], Andrey V. Kartashov[1*], Chong Liu[1*], Hiromi Imamichi[4], Wenjing Yang[5], Weiqun Peng[5], H. Clifford Lane[4], Keji Zhao[6#]

Contact Information

Artem Barski
Divisions of Allergy & Immunology and Human Genetics
Cincinnati Children's Hospital Medical Center
University of Cincinnati College of Medicine
S.6.409 MLC7028
3333 Burnet Avenue,
Cincinnati, OH 45229-3039
Phone: (513) 636-1851
Fax: (513) 636-3310
artem.barski@cchmc.org

Keji Zhao
Systems Biology Center
National Heart, Lung and Blood Institute
National Institutes of Health
Bldg 10; Room 7B05
9000 Rockville Pike
Bethesda, MD 20892
Phone: 301 496 2098
Fax: 301 480 0961
Email: zhaok@nhlbi.nih.gov

## Figures S1-S7 and Table S1.

## Table S2 is provided as a separate Excel file.

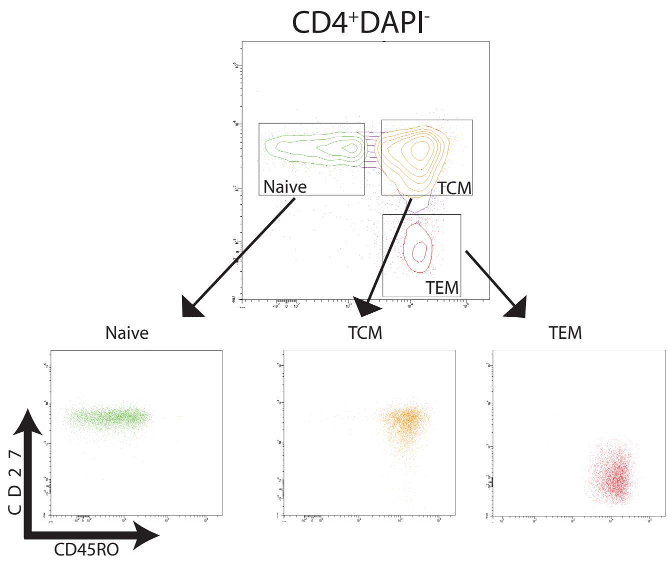


**Figure S1. Experimental approach.** Total human CD4 T cells were sorted into Naïve T cells ($CD4^+CD45RO^-CD27^+$), TCM ($CD4^+CD45RO^+CD27^+$) and TEM ($CD4^+CD45RO^+CD27^-$) subsets.

Figure S2. Chromatin environment at IFNG (a) and IL4/13 (b) loci. Levels of H2A.Z and H3K4me1/3 are shown in Naïve, CM and EM T cells, as well as H7ESC cells, CD34 HSC cells and CD 20 B cells. CD34 data are from ref20 whereas, CD20 and H7 data are from ENCODE[26] project (Bernstein lab at Broad Institute and Stamatoyannopoulos lab at UW). Total resting and activated CD4 data are from refs. 13 and 11. Th1 and Th2 data are from ref. 27.

a

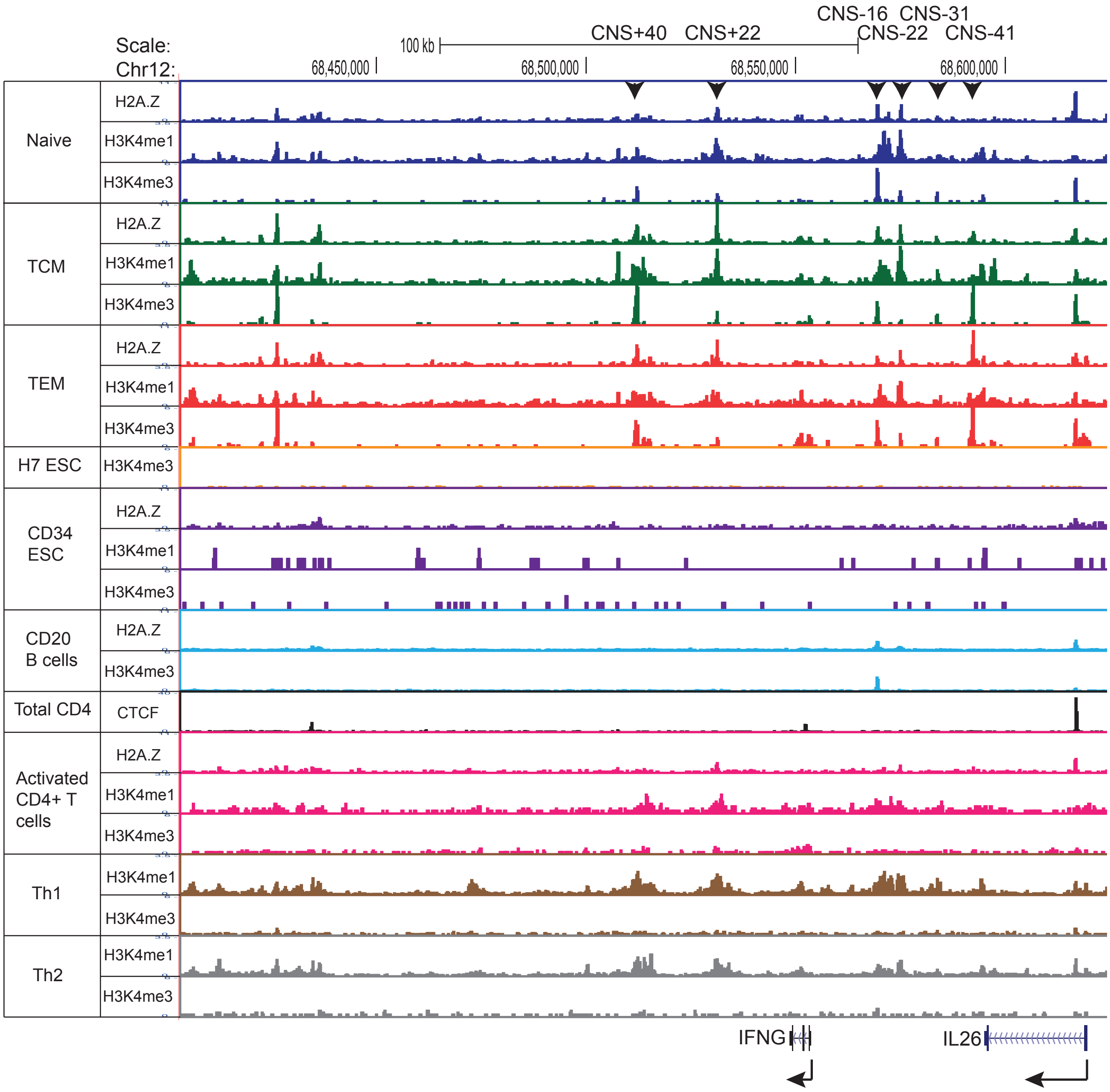

Figure S2. Chromatin environment at IFNG (a) and IL4/13 (b) loci. Levels of H2A.Z and H3K4me1/3 are shown in Naïve, CM and EM T cells, as well as H7ESC cells, CD34 HSC cells and CD 20 B cells. CD34 data are from ref20 whereas, CD20 and H7 data are from ENCODE[26] project (Bernstein lab at Broad Institute and Stamatoyannopoulos lab at UW). Total resting and activated CD4 data are from refs. 13 and 11. Th1 and Th2 data are from ref. 27.

b

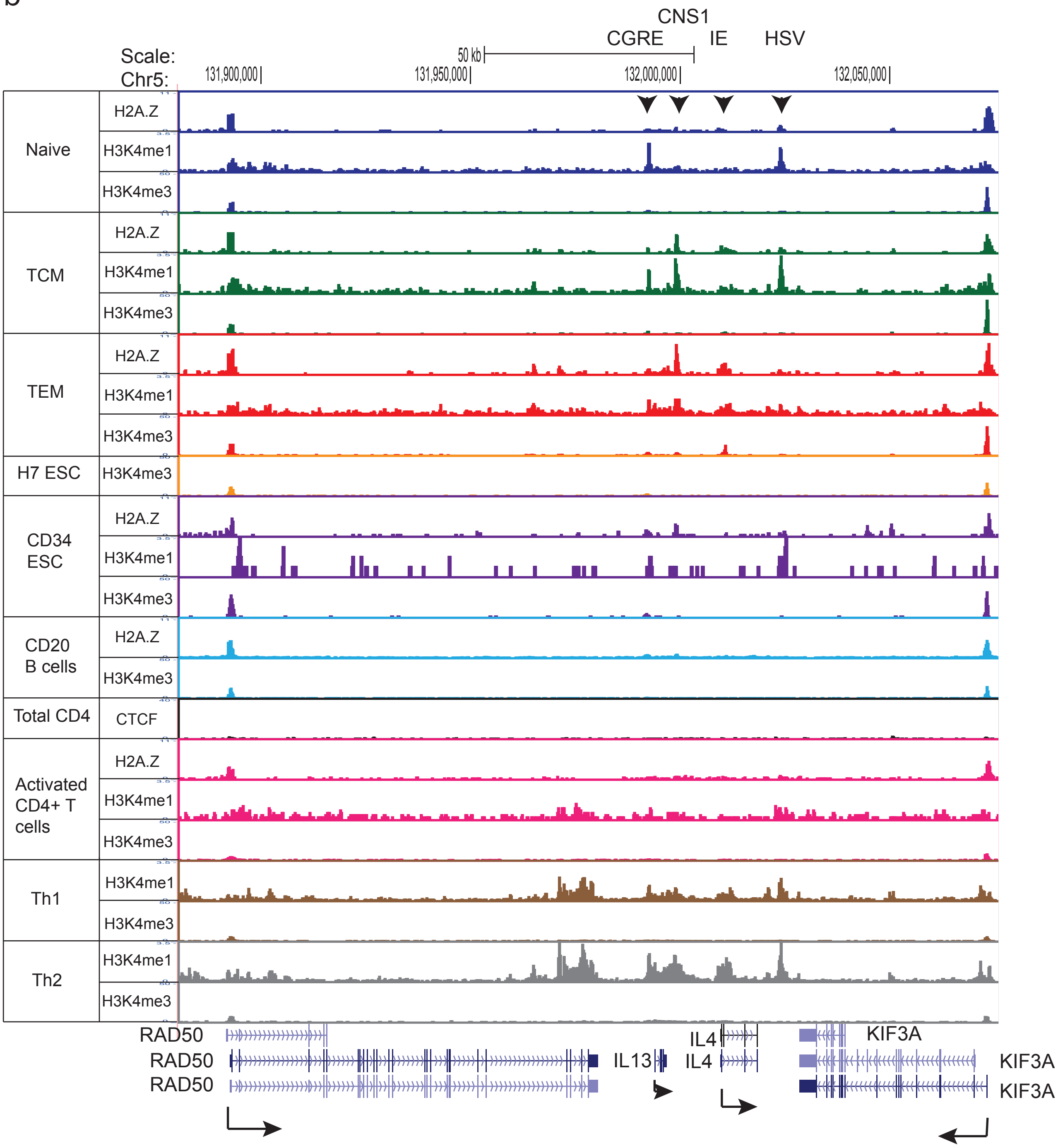

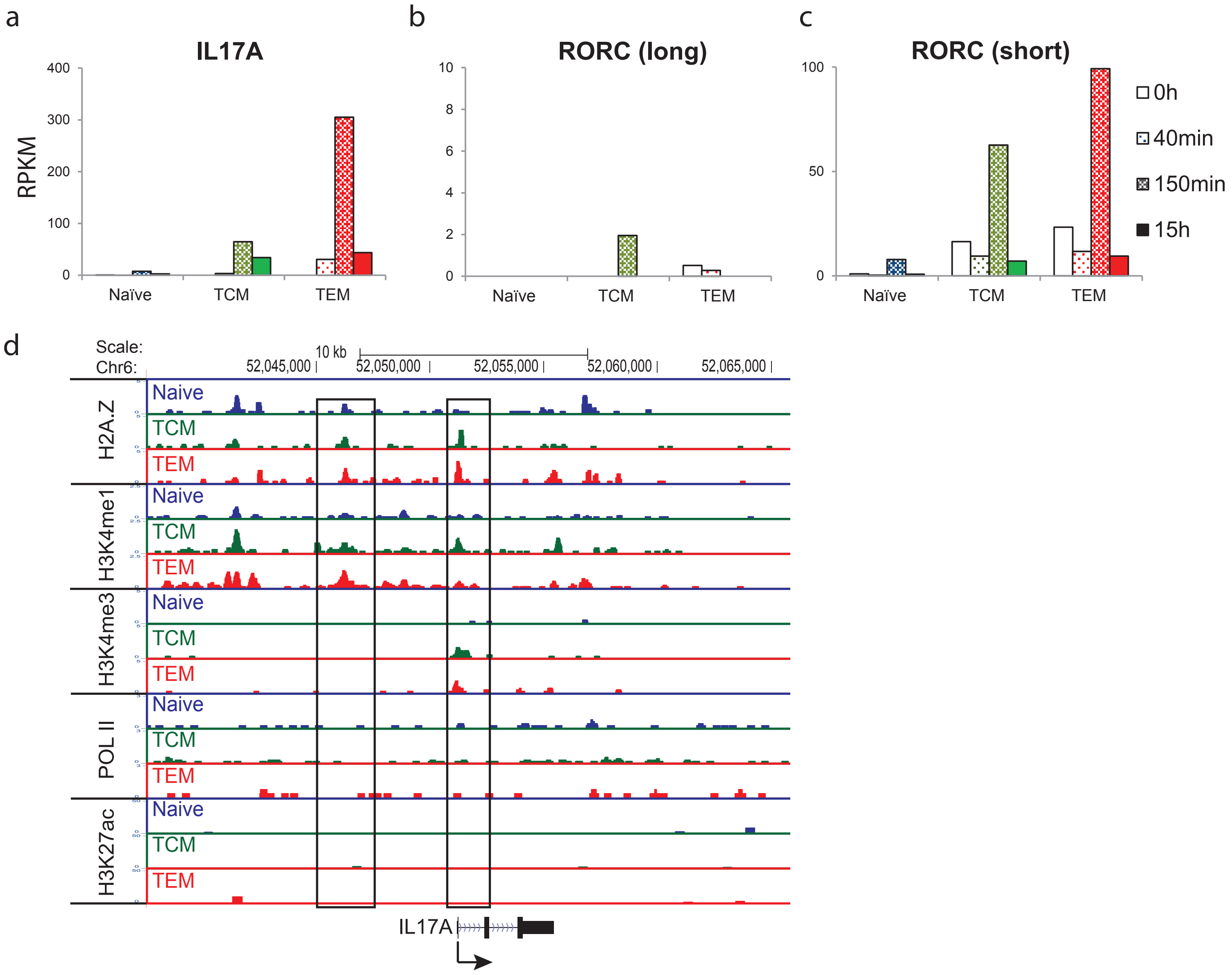


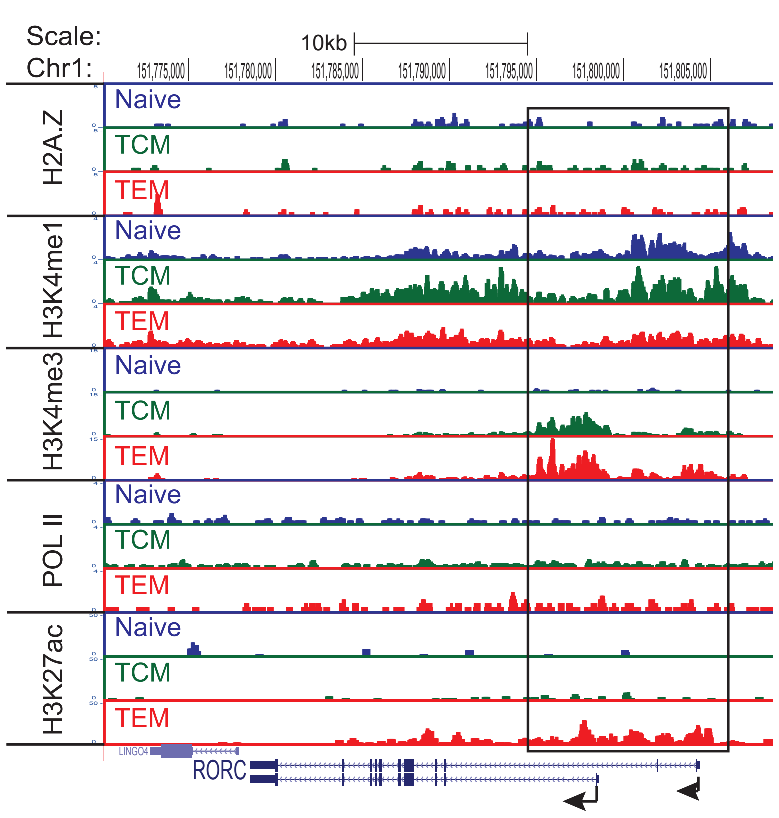


**Figure S3. Gene expression and chromatin environment at selected loci.** IL17A (a,d), RORC (b,c,e) GATA3 (f,h) TBX21 (g,i ), IL9(j,l), IL22 (k,m), IL3 (n,p), CSF2 (o,p) and, housekeepeing genes, PPIA (q), EEF1A1(r) and B2M (s). Chromatin environment is shown in resting cells and gene expression is shown in resting cells and cells activated for 40 min, 150 min or 15h, as measured by RNA-Seq.

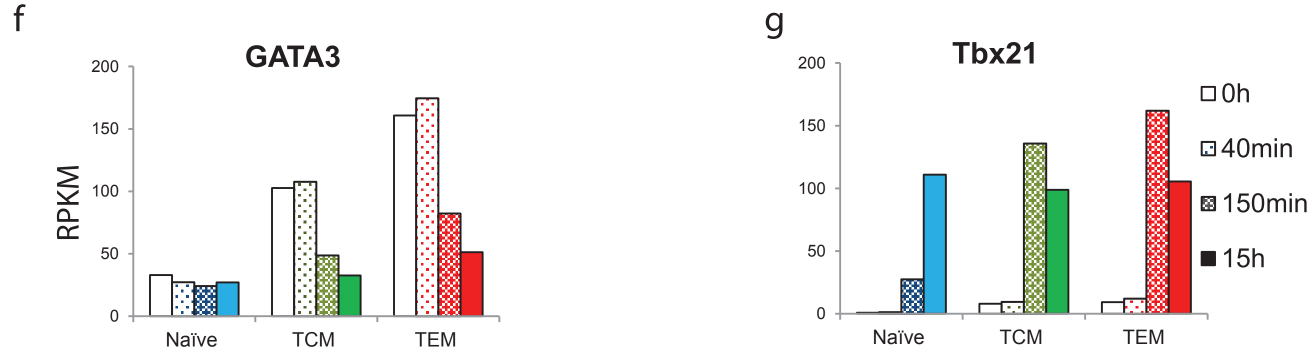


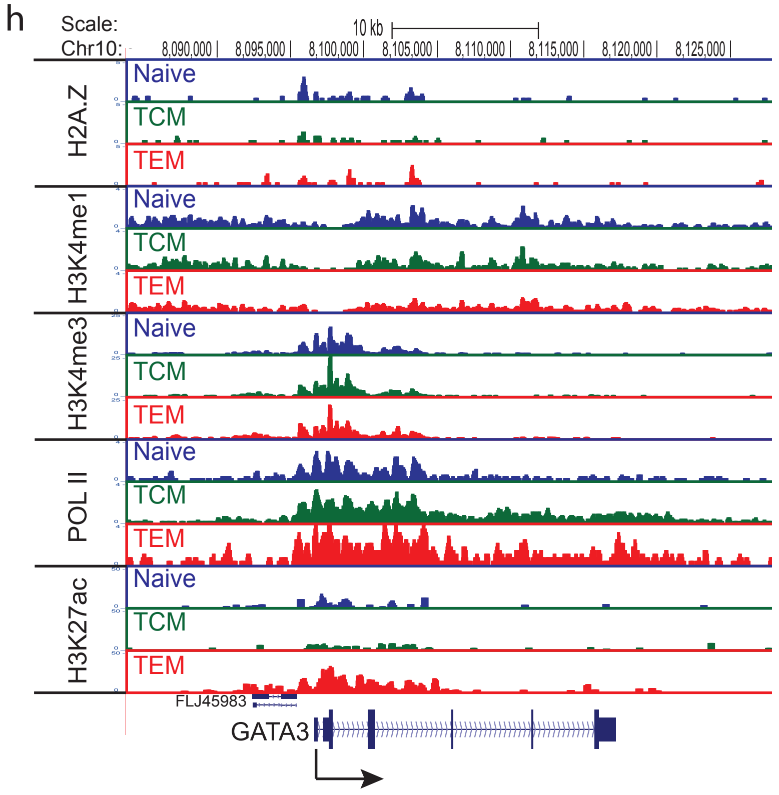


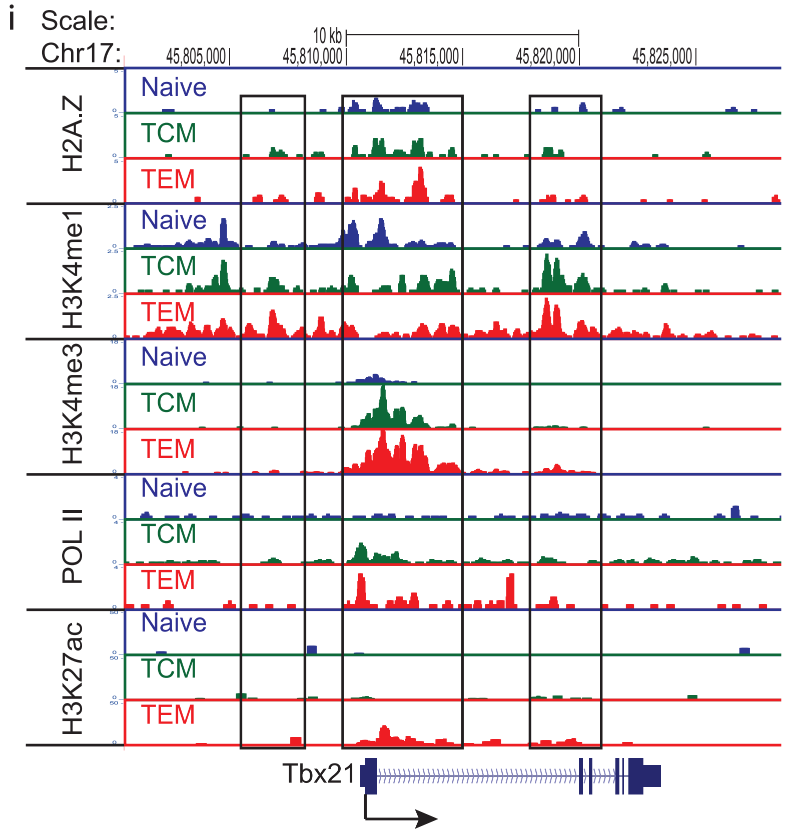


**Figure S3. Gene expression and chromatin environment at selected loci.** IL17A (a,d), RORC (b,c,e) GATA3 (f,h) TBX21 (g,i ), IL9(j,l), IL22 (k,m), IL3 (n,p), CSF2 (o,p) and, housekeepeing genes, PPIA (q), EEF1A1(r) and B2M (s). Chromatin environment is shown in resting cells and gene expression is shown in resting cells and cells activated for 40 min, 150 min or 15h, as measured by RNA-Seq.

j

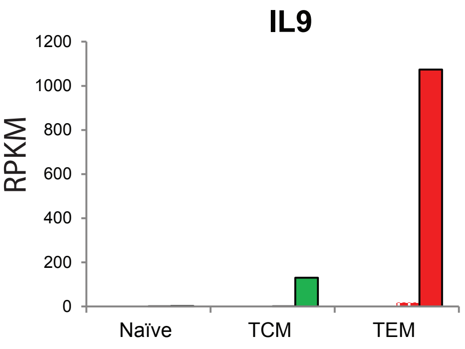


k

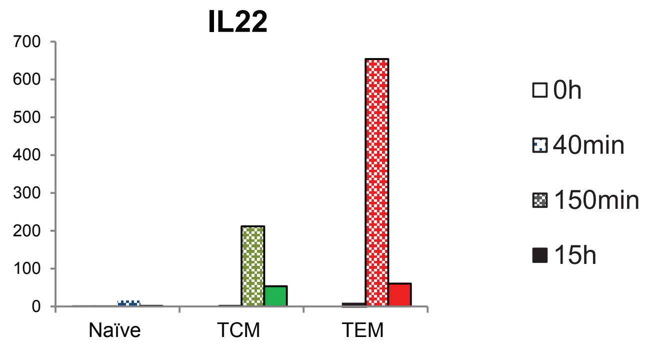


l

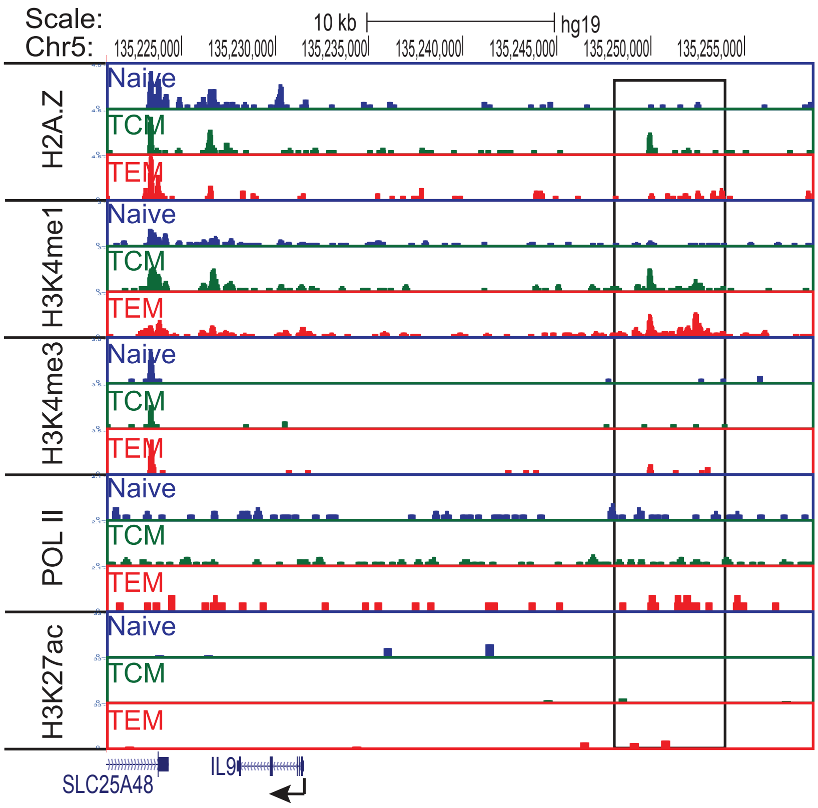


m

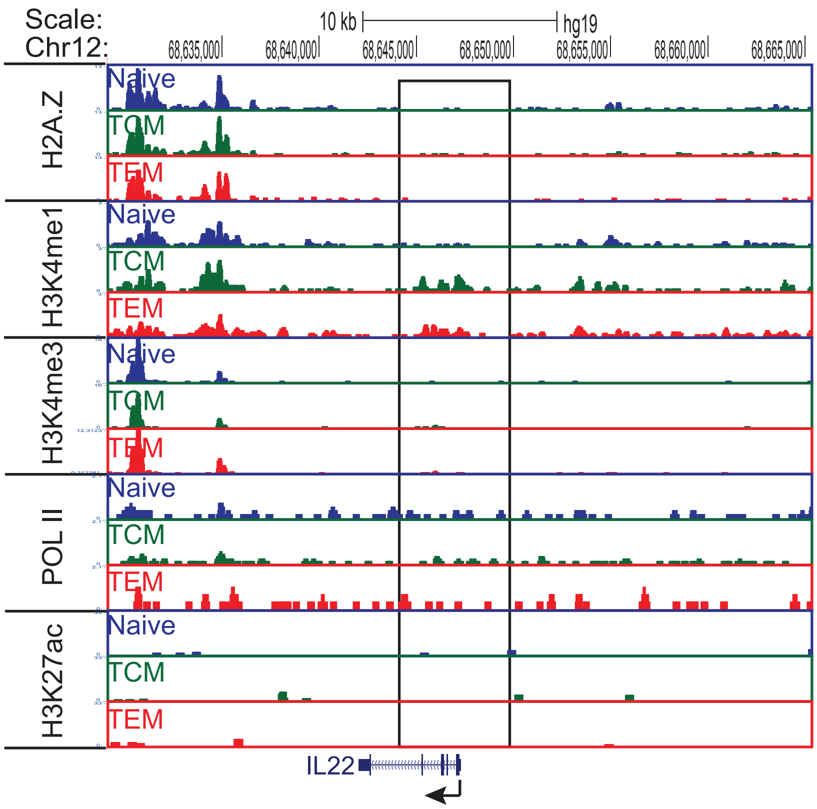


**Figure S3. Gene expression and chromatin environment at selected loci.** IL17A (a,d), RORC (b,c,e) GATA3 (f,h) TBX21 (g,i ), IL9(j,l), IL22 (k,m), IL3 (n,p), CSF2 (o,p) and, housekeepeing genes, PPIA (q), EEF1A1(r) and B2M (s). Chromatin environment is shown in resting cells and gene expression is shown in resting cells and cells activated for 40 min, 150 min or 15h, as measured by RNA-Seq.

n

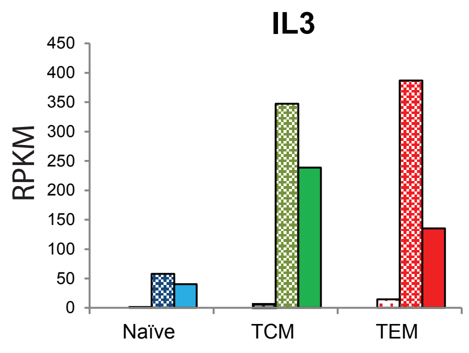


o

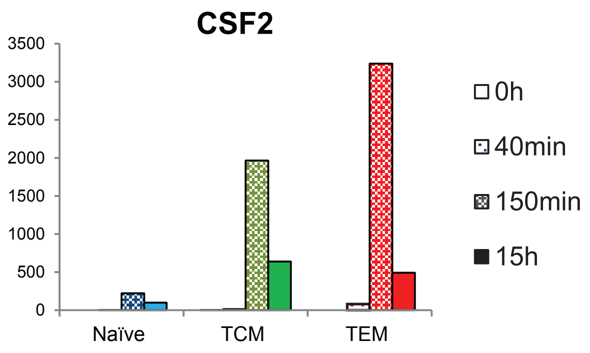


p

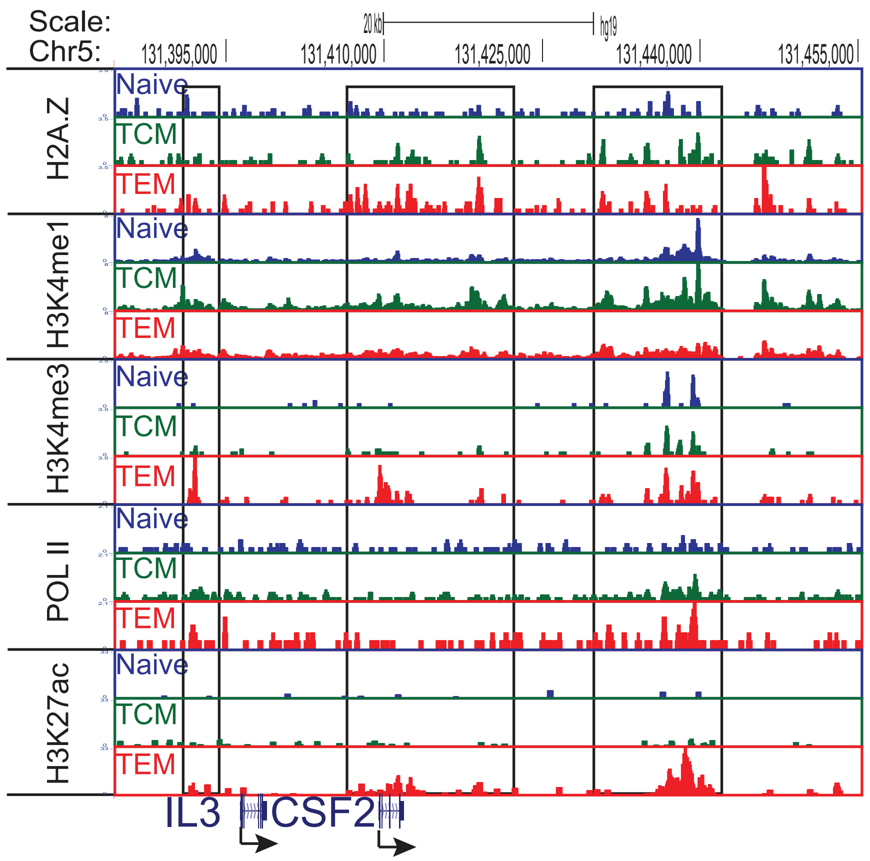


**Figure S3. Gene expression and chromatin environment at selected loci.** IL17A (a,d), RORC (b,c,e) GATA3 (f,h) TBX21 (g,i ), IL9(j,l), IL22 (k,m), IL3 (n,p), CSF2 (o,p) and, housekeepeing genes, PPIA (q), EEF1A1(r) and B2M (s). Chromatin environment is shown in resting cells and gene expression is shown in resting cells and cells activated for 40 min, 150 min or 15h, as measured by RNA-Seq.

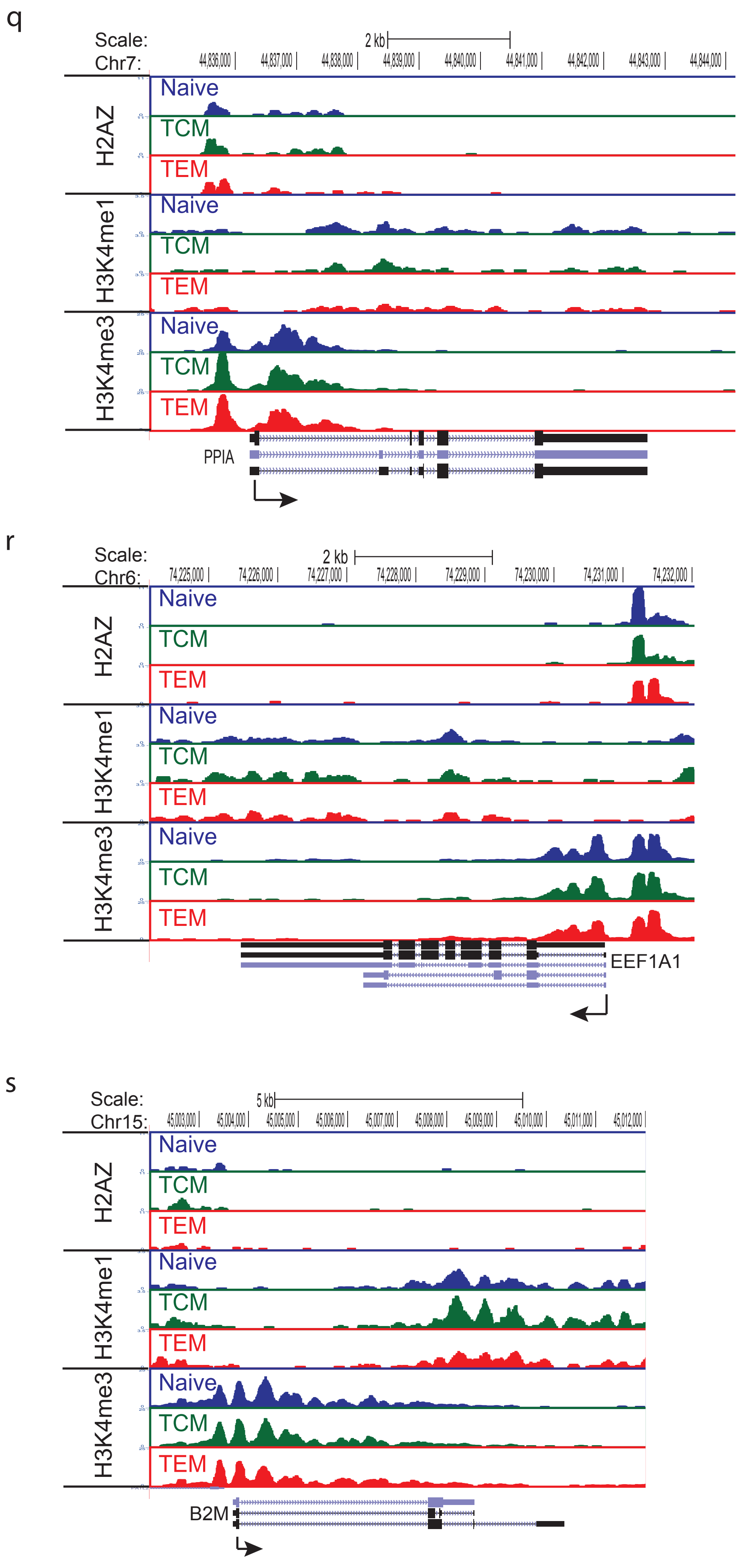


**Figure S3. Gene expression and chromatin environment at selected loci.** IL17A (a,d), RORC (b,c,e) GATA3 (f,h) TBX21 (g,i ), IL9(j,l), IL22 (k,m), IL3 (n,p), CSF2 (o,p) and, housekeepeing genes, PPIA (q), EEF1A1(r) and B2M (s). Chromatin environment is shown in resting cells and gene expression is shown in resting cells and cells activated for 40 min, 150 min or 15h, as measured by RNA-Seq.

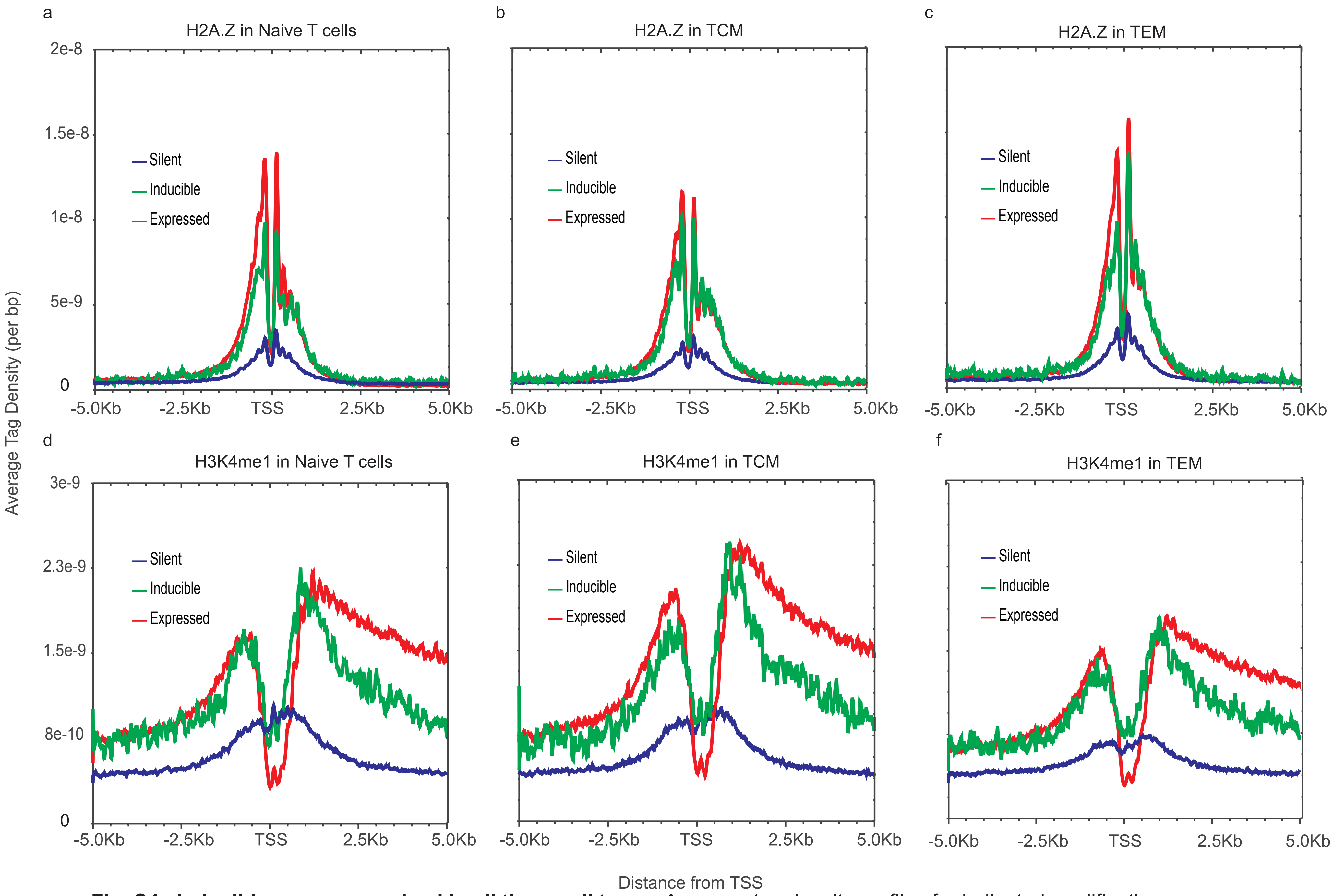


**Fig. S4. Inducible genes are poised in all three cell types.** Average tag density profiles for indicated modifications were built for genes that are expressed in resting cells (E), those that are silent in resting cells (S) and those that are silent in resting cells, but become induced upon activation (I). Distribution of expression values for the groups of genes is shown in panel (x).

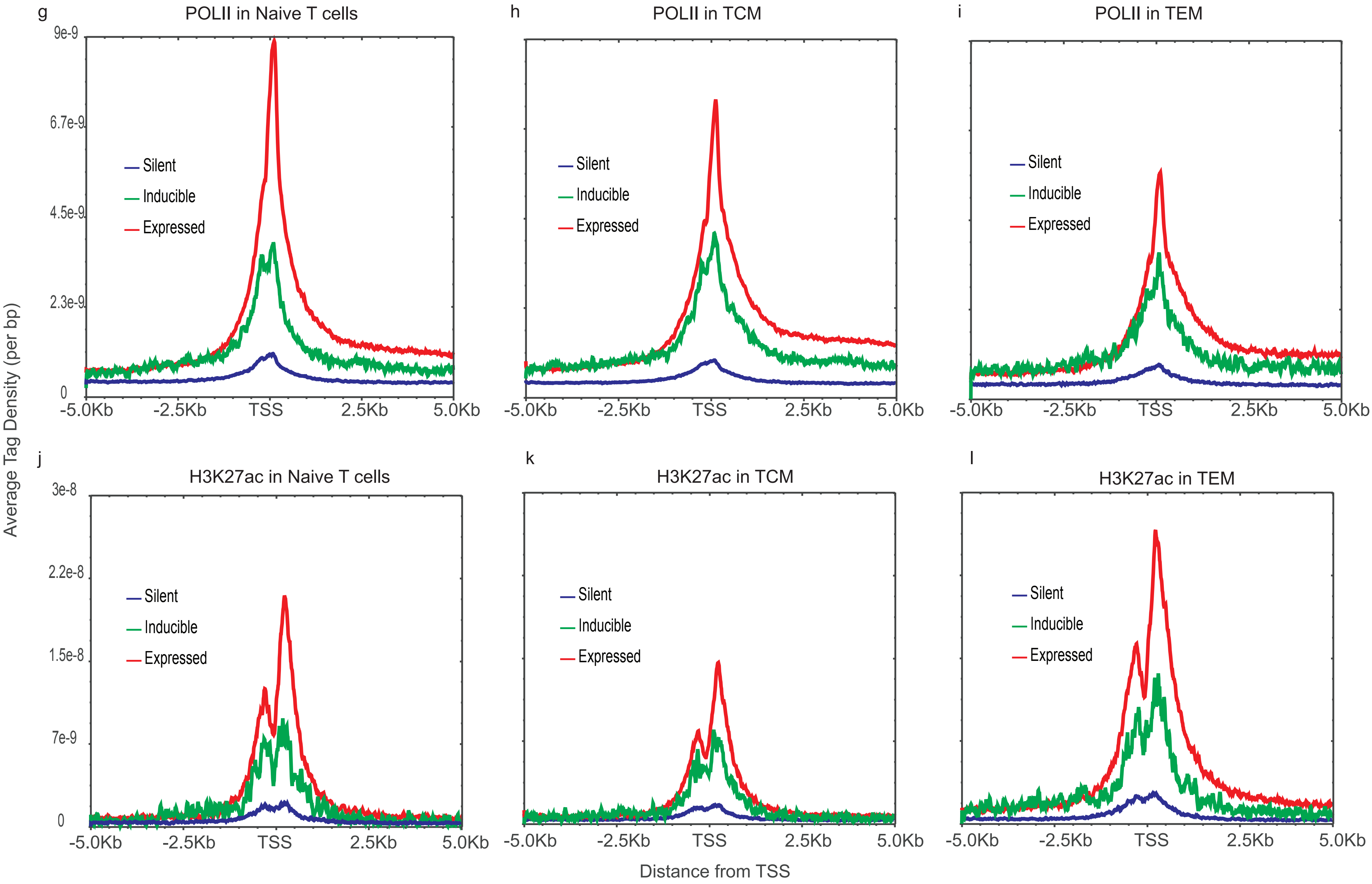


**Fig. S4. Inducible genes are poised in all three cell types.** Average tag density profiles for indicated modifications were built for genes that are expressed in resting cells (E), those that are silent in resting cells (S) and those that are silent in resting cells, but become induced upon activation (I). Distribution of expression values for the groups of genes is shown in panel (x).

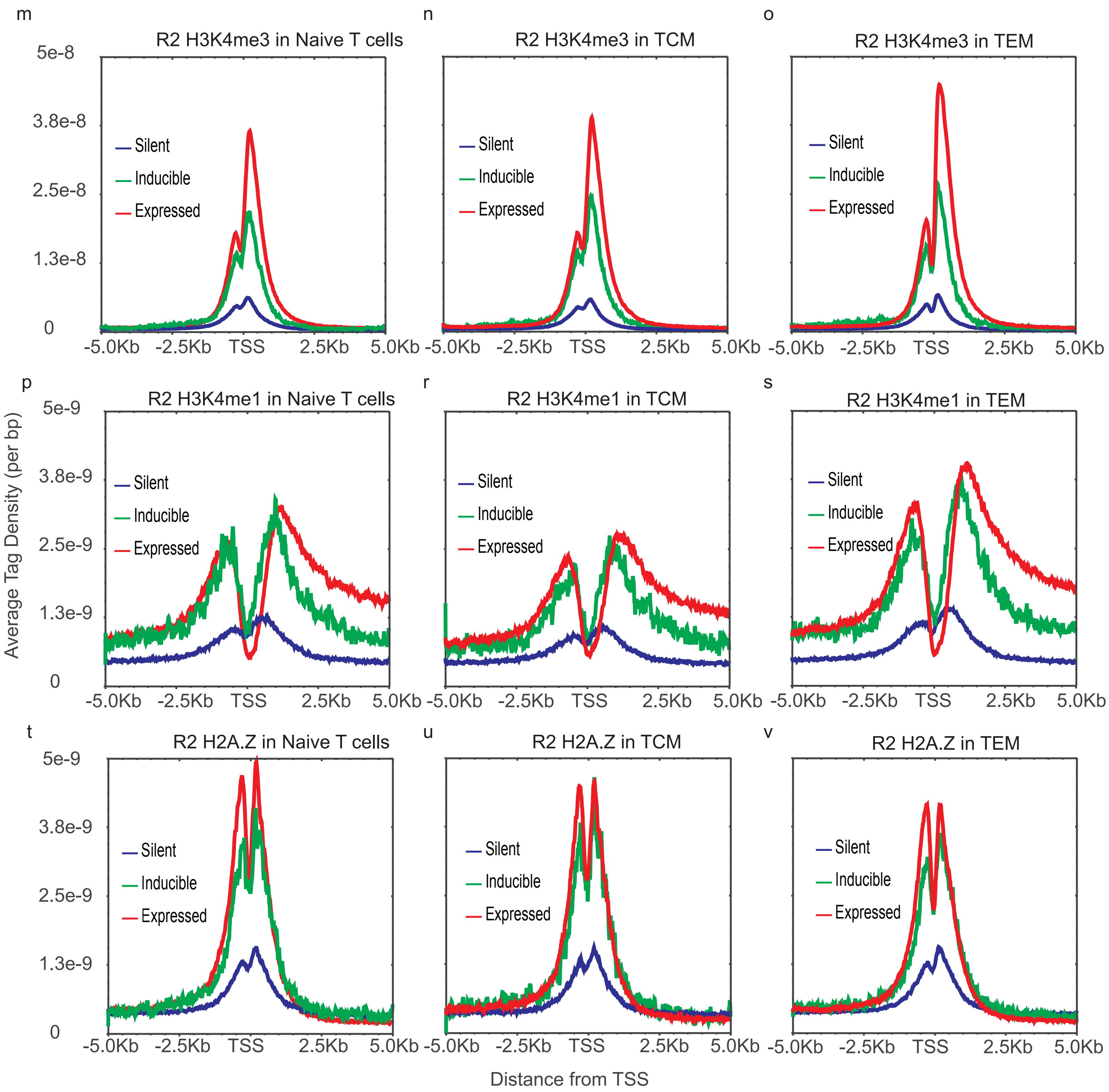


**Fig. S4. Inducible genes are poised in all three cell types.** Average tag density profiles for indicated modifications were built for genes that are expressed in resting cells (E), those that are silent in resting cells (S) and those that are silent in resting cells, but become induced upon activation (I). Distribution of expression values for the groups of genes is shown in panel (x).

x

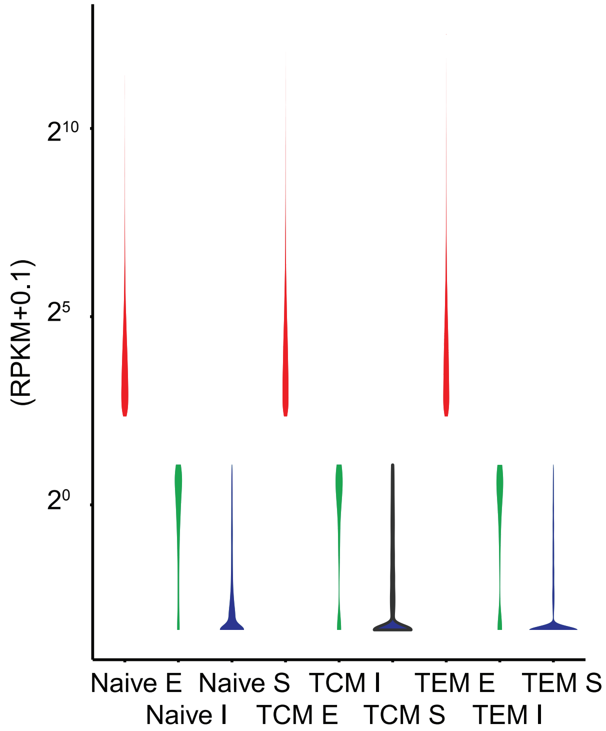


**Fig. S4. Inducible genes are poised in all three cell types.** Average tag density profiles for indicated modifications were built for genes that are expressed in resting cells (E), those that are silent in resting cells (S) and those that are silent in resting cells, but become induced upon activation (I). Distribution of expression values for the groups of genes is shown in panel (x).

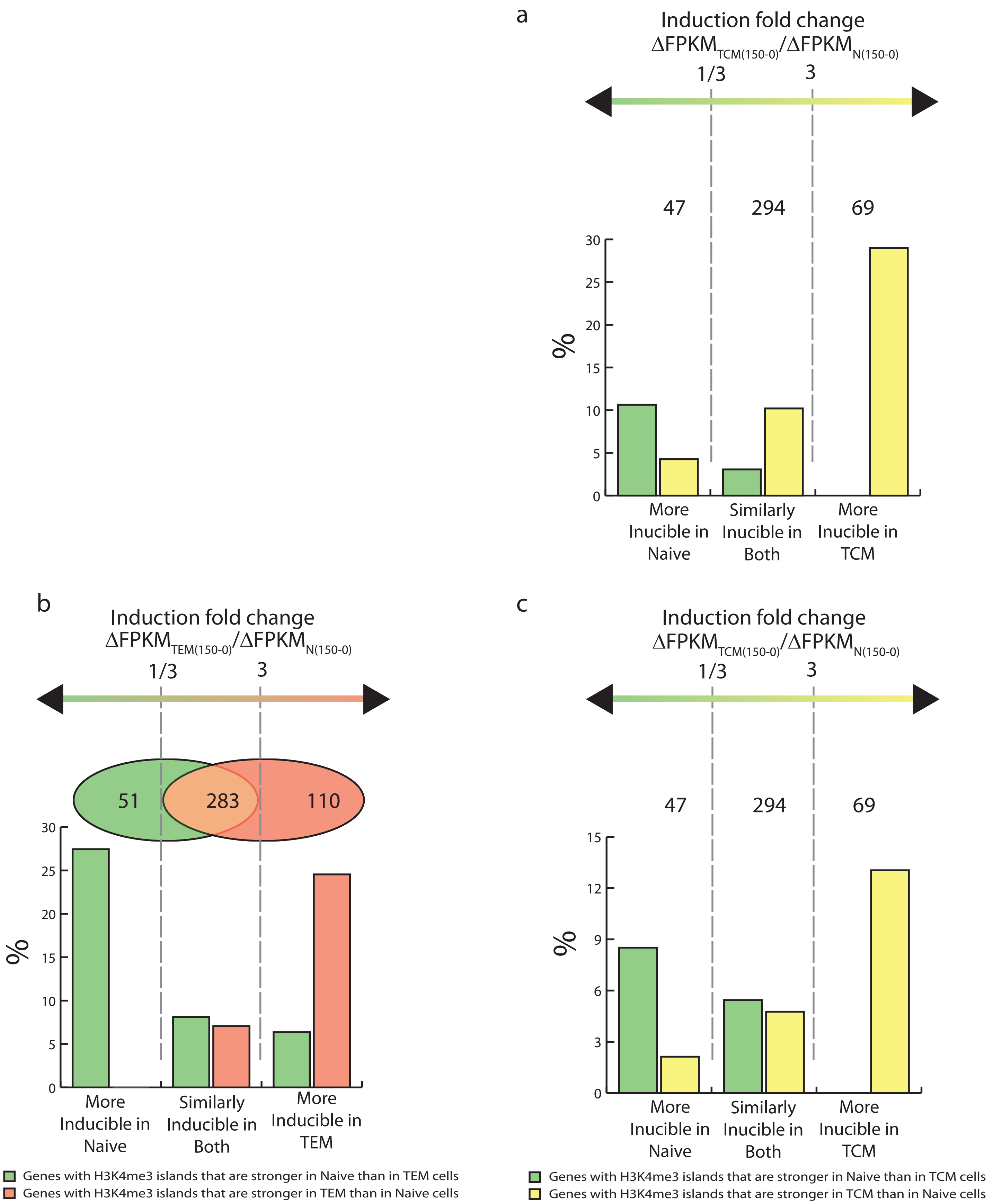


**Figure S5. Differential gene inducibility can be explained by chromatin state. (a)** Genes that were induced upon activation in either naive or cental memory T cells were categorized into 3 groups: more inducible in Naïve, more inducible in TCM or inducible in both (as shown by Venn diagram). Bar graphs show the percentage of genes in each category that have gained or lost H3K4me3 in their vicinity [-20kb from TSS, +1 kb from TSS] during transition from naïve to memory state. **(b, c)** Same as Fig. 3 and Fig. S5a, but based on the second replicate data.

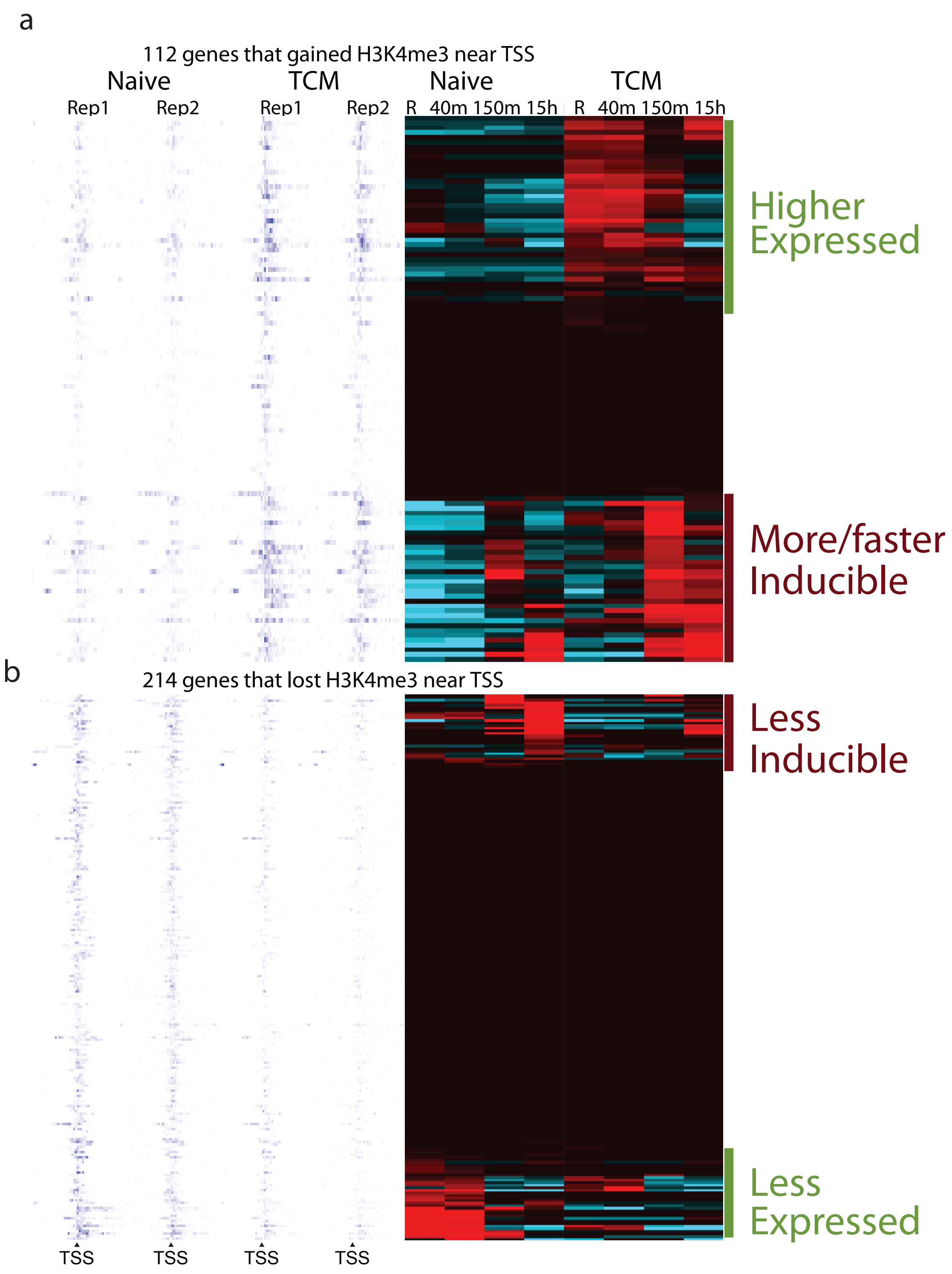


**Figure S6. Gain of H3K4me3 at promoter may lead to increased expression and/or inducibility, whereas loss of H3K4me3 may lead to loss of expression and/or inducibility.** On the left tag density heatmaps show the level of H3K4me3 within 5kb around TSS for genes that gained (a) or lost (b) H3K4me3 islands at the promoter in the transition from naïve T cells (Naïve) to central memory T cells (TCM). On the right heatmaps show level of expression for the corresponding genes in resting cells (R) and cells activated for the period shown.

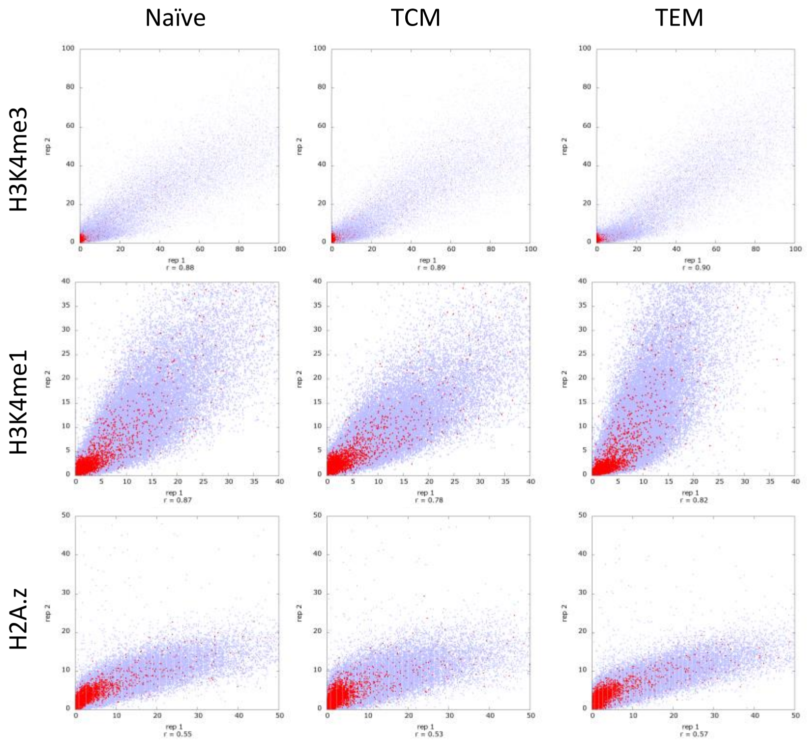


**Figure S7. Correlation between replicates.** The genome was separated into 10kb bins and the normalized number of reads in each window was plotted for replicate 1 against replicate 2. Pearson R-value is shown under each plot.

Supplementary table 1a. Gene ontology analysis of 188 genes that *gain* H3K4me3 positive modification at their promoters in transition from naïve to EM T cells. Top 25 terms from GO-biological process category are shown.

| **ID** | **Name** | **p-value** |
|---|---|---|
| GO:0001775 | cell activation | 1.22E-09 |
| GO:0070231 | T cell apoptotic process | 3.02E-09 |
| GO:0040011 | locomotion | 1.76E-08 |
| GO:0034109 | homotypic cell-cell adhesion | 2.31E-08 |
| GO:0006935 | chemotaxis | 5.00E-08 |
| GO:0030334 | regulation of cell migration | 5.11E-08 |
| GO:0042330 | taxis | 5.30E-08 |
| GO:0040012 | regulation of locomotion | 5.91E-08 |
| GO:0040017 | positive regulation of locomotion | 5.92E-08 |
| GO:2001237 | negative regulation of extrinsic apoptotic signaling pathway | 7.20E-08 |
| GO:1903037 | regulation of leukocyte cell-cell adhesion | 7.68E-08 |
| GO:0070227 | lymphocyte apoptotic process | 7.69E-08 |
| GO:0030155 | regulation of cell adhesion | 8.34E-08 |
| GO:0045321 | leukocyte activation | 9.84E-08 |
| GO:0051249 | regulation of lymphocyte activation | 1.08E-07 |
| GO:0007159 | leukocyte cell-cell adhesion | 1.08E-07 |
| GO:0050865 | regulation of cell activation | 1.15E-07 |
| GO:0050867 | positive regulation of cell activation | 1.28E-07 |
| GO:0006955 | immune response | 1.28E-07 |
| GO:0002682 | regulation of immune system process | 1.40E-07 |
| GO:0042110 | T cell activation | 1.49E-07 |
| GO:0070489 | T cell aggregation | 1.49E-07 |
| GO:0071593 | lymphocyte aggregation | 1.53E-07 |
| GO:0002694 | regulation of leukocyte activation | 1.68E-07 |
| GO:2000145 | regulation of cell motility | 1.75E-07 |

Supplementary table 1b. Gene ontology analysis of 436 genes that *lose* H3K4me3 positive modification at their promoters in transition from naïve to EM T cells. Top 25 terms from GO-biological process category are shown.

| ID | Name | p-value |
|---|---|---|
| GO:2000026 | regulation of multicellular organismal development | 2.74E-07 |
| GO:0022008 | neurogenesis | 3.81E-07 |
| GO:0030182 | neuron differentiation | 1.04E-06 |
| GO:0009887 | organ morphogenesis | 1.88E-06 |
| GO:0038007 | netrin-activated signaling pathway | 3.07E-06 |
| GO:0006928 | movement of cell or subcellular component | 3.67E-06 |
| GO:0048699 | generation of neurons | 3.90E-06 |
| GO:0007416 | synapse assembly | 3.95E-06 |
| GO:0048667 | cell morphogenesis involved in neuron differentiation | 4.91E-06 |
| GO:0048812 | neuron projection morphogenesis | 6.90E-06 |
| GO:0051094 | positive regulation of developmental process | 8.80E-06 |
| GO:0016477 | cell migration | 1.13E-05 |
| GO:0040008 | regulation of growth | 1.65E-05 |
| GO:0048870 | cell motility | 1.80E-05 |
| GO:0051674 | localization of cell | 1.80E-05 |
| GO:0048638 | regulation of developmental growth | 1.89E-05 |
| GO:0048729 | tissue morphogenesis | 2.21E-05 |
| GO:0051270 | regulation of cellular component movement | 2.30E-05 |
| GO:0001525 | angiogenesis | 2.72E-05 |
| GO:0001944 | vasculature development | 2.89E-05 |
| GO:0030334 | regulation of cell migration | 3.05E-05 |
| GO:0000904 | cell morphogenesis involved in differentiation | 3.16E-05 |
| GO:0001568 | blood vessel development | 3.36E-05 |
| GO:0045664 | regulation of neuron differentiation | 3.77E-05 |
| GO:0051960 | regulation of nervous system development | 3.87E-05 |
| GO:0036301 | macrophage colony-stimulating factor production | 3.90E-05 |
